# MicroRNA-223 inhibits neutrophil extracellular traps formation through regulating calcium influx and small extracellular vesicles transmission

**DOI:** 10.1038/s41598-021-95028-0

**Published:** 2021-08-03

**Authors:** Tsai-Ling Liao, Yi-Ming Chen, Kuo-Tung Tang, Po-Ku Chen, Hung-Jen Liu, Der-Yuan Chen

**Affiliations:** 1 Department of Medical Research, Taichung Veterans General Hospital, Taichung, 407 Taiwan; 2 Rong Hsing Research Center for Translational Medicine, National Chung Hsing University, Taichung, 402 Taiwan; 3 Ph.D. Program in Translational Medicine, National Chung Hsing University, Taichung, 402 Taiwan; 4 Division of Allergy, Immunology and Rheumatology, Taichung Veterans General Hospital, Taichung, 407 Taiwan; 5 Rheumatology and Immunology Center, Department of Medicine, China Medical University Hospital, No. 2, Yude Road, Taichung, 40447 Taiwan; 6 Translational Medicine Laboratory, Rheumatology and Immunology Center, China Medical University Hospital, Taichung, 404 Taiwan; 7 College of Medicine, China Medical University, Taichung, 404 Taiwan; 8 Institute of Molecular Biology, National Chung Hsing University, Taichung, 402 Taiwan; 9 The iEGG and Animal Biotechnology Center, National Chung Hsing University, Taichung, 402 Taiwan; 10 Institute of Medicine, Chung Shan Medical University Hospital, Taichung, 402 Taiwan

**Keywords:** Cell death and immune response, Autoinflammatory syndrome

## Abstract

Modulation of miRNAs and neutrophil extracellular traps (NETs) formation are both implicated in inflammatory disorders. Adult-onset Still’s disease (AOSD) is a systemic autoinflammatory disease with neutrophilic leukocytosis and unknown etiology. Although the NETs formation is elevated in AOSD patients, the regulatory roles of miRNAs in NETs formation in AOSD remains unclear. We revealed that the circulating levels of IL-18, NETs, and miR-223 were significantly higher in active AOSD patients, compared with inactive AOSD patients or healthy controls (*P* < 0.005). Moreover, IL-18 increased calcium influx into neutrophils, which led to mitochondrial ROS (mROS) production and NETs formation. Elevated levels of NETs-DNA could induce miR-223 expression in neutrophils through activating Toll-like receptor 9. The upregulated miR-223 expression in neutrophils suppressed mROS production by blocking calcium influx, and subsequently inhibited IL-18-mediated NETs formation. Besides, the increased neutrophil-derived exosomal miR-223 levels were observed in active AOSD patients compared with healthy controls (*P* < 0.005). Our in vitro assays demonstrated that the neutrophil-derived small extracellular vesicles carried miR-223, which could repress IL-18 production in macrophages. Together, these results suggest a fine-tuned mechanism between inflammatory (IL-18 induced NETs) and anti-inflammatory (miR-223) factors in AOSD. MiR-223, mROS inhibitors, and calcium channel blockers are the potential therapeutics for autoinflammatory diseases such as AOSD.

## Introduction

Neutrophils have a variety of important biological functions in both innate and adaptive immunities, thus playing a key role in inflammation^1,2^. A novel feature of neutrophil biology is its ability to generate neutrophil extracellular traps (NETs)^3^. Adult onset Still's disease (AOSD) is an auto-inflammatory disorder characterized by fever, rash, arthritis, and neutrophilic leukocytosis^4^. Accumulating evidence reveals that an elevated NETs was observed in AOSD patients, and circulating NETs levels significantly declined in parallel with disease remission^5,6^. Recently, we observed that IL-18 could enhance NETs formation in healthy neutrophils and the differentiated HL-60 human neutrophil-like (dHL-60) cells (unpublished manuscript), but the regulatory mechanism is unclear.

On the other hand, microRNAs (miRNAs) are endogenous noncoding RNAs that post-transcriptionally repress the expression of target messenger RNAs (mRNAs)^7^. MiRNAs have been implicated in modulating host immune response^8^. Previously, we observed that miR-223 is upregulated in AOSD patients by using microarray analysis^9^. MiR-223 is abundantly expressed in neutrophils where it plays a crucial role in regulating granulopoiesis and neutrophil function^10^. Small extracellular vesicles (sEVs) released by cells that could carry proteins and nucleic acids (e.g., miRNAs) to contribute intercellular communication and regulate biological function^11^. Shao et al.^12^ demonstrated that neutrophil-derived sEVs enhance the skin auto-inflammation via activating keratinocytes. Moreover, it had been reported that activated neutrophil-derived sEVs could degrade tight junctions to disrupt epithelial barriers^13^. But the role of sEVs either in NETs formation in neutrophils or auto-inflammation in AOSD is still uncertain.

Given the augmented effects of AOSD-related inflammatory cytokines on NETs formation and the potential role of miR-223 in neutrophils, we hypothesize that IL-18 and miR-223 may be involved in NETs formation in AOSD. In this pilot study, we aimed to explore the regulatory roles of IL-18 and miR-223 in the NETs formation of AOSD by using an in vitro cell-based assay.

## Results

### Clinical characteristics of AOSD patients

Among the 38 active AOSD patients (median of activity scores 6.0, range 4–9), spiking fever (≥ 39 °C), rash, arthralgia or arthritis, sore throat, liver dysfunction, and lymphadenopathy were noted in 37 (97.4%), 34 (89.5%), 32 (84.2%), 27 (71.1%), 23 (60.5%), and 17 (44.7%) patients, respectively. There were no significant differences in age at study entry or the proportion of females between AOSD patients and healthy subjects (Table 1).

**Table 1:** Demographic data and laboratory findings of patients with Adult-onset Still’s disease (AOSD) and healthy controls (HC).

Characteristics	AOSD (n = 38)	HC (n = 26)
Age at study entry, years	37.6 ± 11.1	37.8 ± 8.3
Female proportion, n (%)	29 (76.3%)	19 (73.1%)
Activity score	6.1 ± 1.4	NA
IL-1β levels, pg/ml	2.37 (0.70–4.92)*	1.02 (0.28–2.89)
IL-6 levels, pg/ml	1012.5 (301.1–1265.8)**	437.6 (94.8–1002.8)
IL-18 levels, pg/ml	6710.4 (755.0–16,948.5)***	7.2 (5.8–49.1)
TNF-α levels, pg/ml	132.1 (35.2–290.1)***	6.1 (3.6–37.3)

^#^Data presented as mean ± SD, number (percentage), or median (interquartile range); NA: not applicable; IL: interleukin; TNF-α: tumor necrosis factor-α.

*P < 0.05, ** *P* < 0.005, *** *P* < 0.001, vs. healthy controls, determined by Mann–Whitney U test.

### Increased levels of NETs in AOSD patients is mitochondrial ROS-dependent

**Figure 1:**
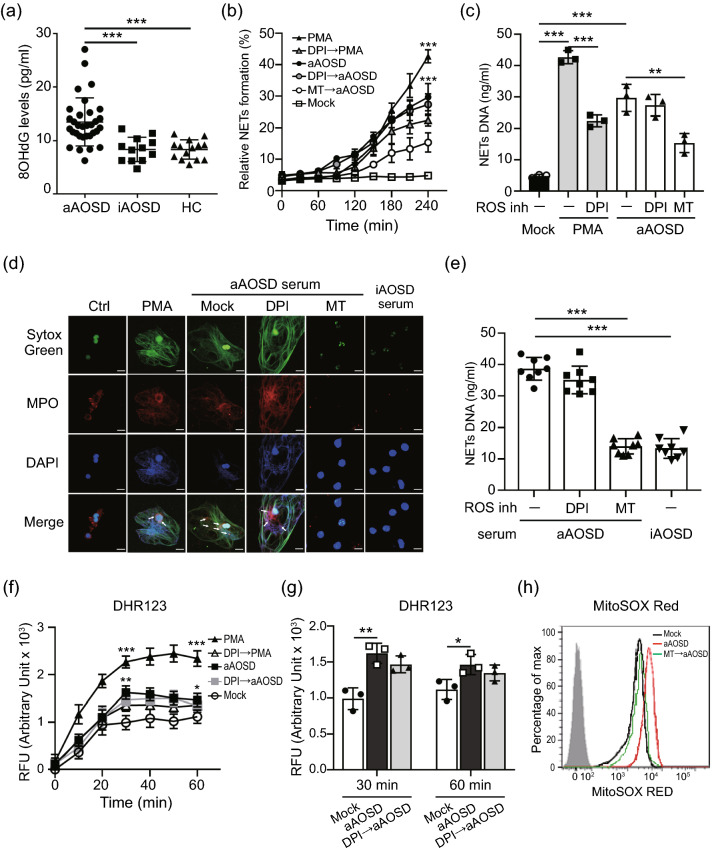
Increased levels of NETs was induced by sera of active AOSD patients is mitochondrial ROS-dependent. (**a**) Increased oxidative stress (8-OHdG) levels in active AOSD patients. (**b**-**d**) Normal human neutrophils were stimulated with PMA, or the serum of active AOSD (aAOSD) patient in the presence or absence of DPI (25 μM) or MitoTEMPO (10 μM). (**b**) The dynamic of NETs formation was analyzed by using Sytox Green with plate reader. The fluorescence readout obtained from cells lysed with 0.5% (vol/vol) Triton X-100 was considered as 100% DNA release, and the relative NETs formation was calculated at each time point. (**c**) The levels of NETs DNA release in the supernatant of cells at 4 h post-treatment was quantified using PicoGreen fluorescence dye with plate reader. (**d**) The NETs formation was detected by confocal microscopy. (**e**) Neutrophils were stimulated with serum of active AOSD (n = 8) or inactive AOSD (n = 8) patients in the presence of the indicated ROS inhibitor and the levels NETs DNA release were quantified. (**f**, **g**) The levels of cytosolic ROS were analyzed using DHR123 with plate reader. (**h**) The levels of mitochondrial ROS were analyzed using MitoSOX Red dye with flow cytometry. All experiments were performed in triplicate, and data are presented as the mean ± SD. **P < 0.01, ***P < 0.001. Scale bars, 5 µm.

Reactive oxygen species (ROS) is essential in regulatory of NETs formation^14^. We observed the levels of oxidative stress (8-OHdG, Fig. 1a) were higher in active AOSD patients compared to inactive AOSD patients or HC. Nicotinamide-adenine dinucleotide phosphate oxidase (NOX) is crucial for major production of ROS. Recent studies demonstrate that mROS generation is related to inflammatory and autoimmune diseases pathogenesis^15^. To explore whether NOX and mROS contributed to NETs formation in AOSD patients, we stimulated normal human neutrophils with the sera of active AOSD patients, in the presence or absence of NOX/mROS inhibitors, then measured the kinetics of NET release by using plate reader assay. PMA is the NOX-dependent NET inducer was used as a positive control. Increased levels of NETs formation were observed in neutrophils, after stimulating with PMA or with the sera of active AOSD patients in a time-dependent manner (*P* < 0.005, Fig. 1b). Additionally, there was no difference in active AOSD patients’ sera-induced NETs formation in the presence of diphenyleneiodonium (DPI, NOX inhibitor), but this dramatically declined in the presence of mitochondria-specific superoxide scavenger MitoTEMPO (*P* < 0.01, Fig. 1c). The immunofluorescence images confirmed that the neutrophils activated with the sera of active AOSD patients release NETs as evidenced by the colocalization of extracellular DNA with myeloperoxidase (MPO) (Fig. 1d). The results were further confirmed by enrolling additional AOSD patients’ sera (n = 8) to repeat the assay, indicating that NETs induced by active AOSD patients’ sera is in an mROS-dependent manner (Fig. 1e).

To analyze the dynamics of ROS generation during AOSD patients' sera-induced NETs, we detected the levels of cytosolic ROS by using a fluorescent indicator DHR123. Elevated levels of cytosolic ROS were induced in neutrophils, after stimulating with PMA or with the sera of active AOSD patients in a time-dependent manner (*P* < 0.005, Fig. 1f). To compare with PMA, the lower levels of cytosolic ROS was produced in cells with AOSD patients' sera stimulation (Fig. 1f). In addition, there was no significantly reduced ROS was revealed in cells with AOSD patients' sera treatment in presence of DPI (Fig. 1g). We further examined the mROS production by using a specific fluorescent indicator MitoSOX Red. Increased mROS was induced in cells after adding AOSD patients' sera, which was declined in the presence of MitoTEMPO (MFI: 4023.0 ± 75.2 vs. 1942.3 ± 138.6, Fig. 1h). Taken together, our results revealed that mROS is crucial for AOSD sera-induced NETs, which is NOX-independent.

### Mitochondrial ROS support IL-18 mediated NETs in AOSD patients

**Figure 2:**
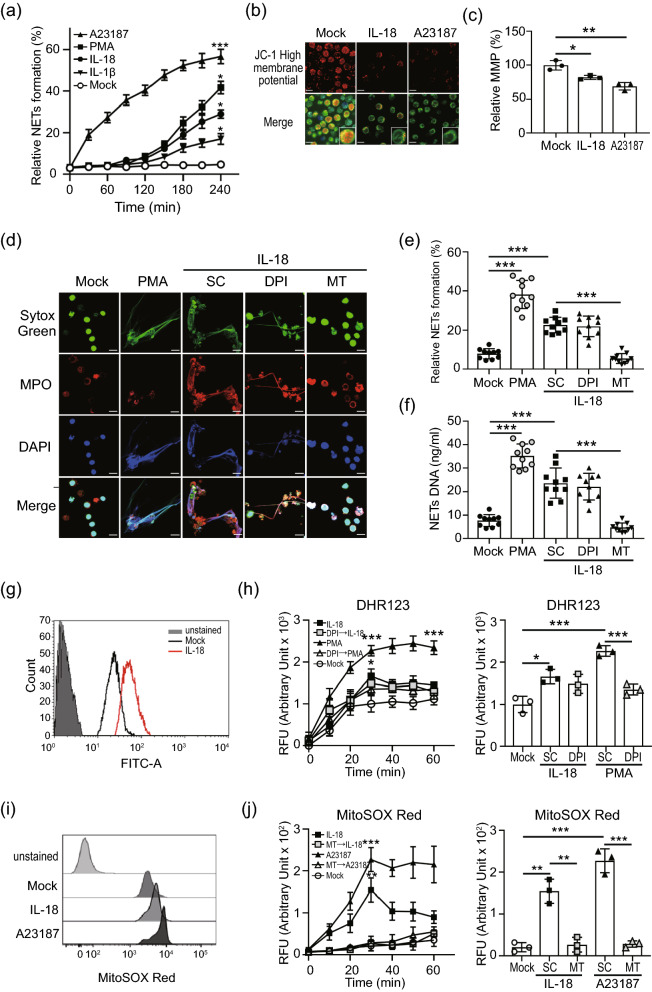
Mitochondrial ROS support IL-18 mediated NETs in AOSD patients. (a) NETs release in response to IL-18 was measured using a plate reader assay. (**b**, **c**) dHL-60 cells were treated with indicated stimulation, (b) the morphology of mitochondria was observed using confocal microscope with MITO-ID dye. (**c**) The mitochondrial membrane potential (MMP) was measured using a plate reader with JC-1 staining. (**d**) Immunofluorescence images of neutrophils were stimulated with IL-18 in the presence of the indicated ROS inhibitor, then stained with Sytox Green (green) and DAPI (blue) to detect DNA, and MPO (red) to detect the NET structure. The PMA treatment was used as a positive control. (**e**, **f**) Neutrophils from AOSD patients (n = 10) which were stimulated with IL-18 in the presence of the indicated ROS inhibitor. The levels of (**e**) NETs formation and (**f**) NETs DNA release were quantified by using plate reader. (**g**) IL-18 induced an elevated cytosolic ROS production in dHL-60 cells. (**h**) The dynamic of cytosolic ROS production in neutrophils was analyzed by using DHR123 dye with plate reader (left panel). At 30 min post-treatment, IL-18 induces a greater cytosolic ROS production compared with control cells (right panel). (**i**) IL-18 induced an elevated mitochondrial ROS production in dHL-60 cells. (**j**) The dynamic of mitochondrial ROS production in neutrophils was analyzed by using MitoSOX Red with plate reader (left panel). At 30 min post-treatment, IL-18 induces a significantly increased mitochondrial ROS production compared with control cells (right panel). All experiments were performed in triplicate, and data are presented as the mean ± SD. The scale bar in the IFA image represents 5 μm. *P < 0.05, **P < 0.01, ***P < 0.005. MT, MitoTEMPO; SC, solvent control.

Given the involvement of IL-1β and IL-18 in AOSD pathogenesis^16,17^, we explored whether IL-1β and/or IL-18 were associated with the increased NETs formation. The results showed a kinetic change in the released NETs DNA from dHL-60 cells treated with IL-1β or IL-18 (Fig. 2a).

Because that the presence of citrullinated histone H3 (citH3) is specific in NOX-independent NETs formation^18^, we examined the induced citH3 levels in cells treated with IL-1β or IL-18. Increased levels of citH3, Akt and ERK activation were seen in cells following IL-18 treatment (Supplementary Fig. S1), suggesting that IL-18-induced NETs occurs in a NOX-independent manner; the same trend was seen for AOSD sera-induced NETs (Fig. 1e). In addition, significantly increased circulating levels of IL-18 were revealed in active AOSD patients (Supplementary Fig. S2) compared with inactive AOSD patients or HC subjects. Based on these observations, we focused on the roles of IL-18 in the NETs of AOSD.

To validate AOSD sera-induced NETs in an mROS-dependent manner is associated with IL-18, we examined the effect of IL-18 on mitochondrial membrane potential (MMP) using MITO-ID dye (Fig. 2b) and tetraethyl benzimidazolyl carbocyanine iodide (JC-1) staining (Fig. 2c). The hypopolarized mitochondria lined the plasma membrane in cells treated with IL-18, in contrast to the evenly distributed hyperpolarized mitochondria in control cells (Fig. 2b). The MMP was decreased in cells after treatment with IL-18 (Fig. 2c). To confirm the associations between ROS and IL-18-mediated NETs in AOSD, neutrophils from inactive AOSD patients (n = 10) were treated with IL-18 in the presence of DPI or MitoTEMPO. It is worth noting that MitoTEMPO markedly suppressed IL-18 mediated NETs (Fig. 2d–f), suggesting that IL-18-induced NETs in AOSD is mROS-dependent.

To investigate whether IL-18-induced NETs is associated with mROS production, we determined total cytosolic ROS and mROS from dHL-60 cells treated with IL-18. Compared to control cells, IL-18 induced the significantly greater production of cytosolic ROS (Fig. 2g–h), and mROS (Fig. 2i–j). In addition, the amount of IL-18-induced mROS was significantly decreased in the presence of MitoTEMPO. Taken together, our results indicated that IL-18 could induce mROS production in neutrophils.

### IL-18 induce NETs formation by increasing calcium influx to regulate mitochondrial ROS generation

**Figure 3:**
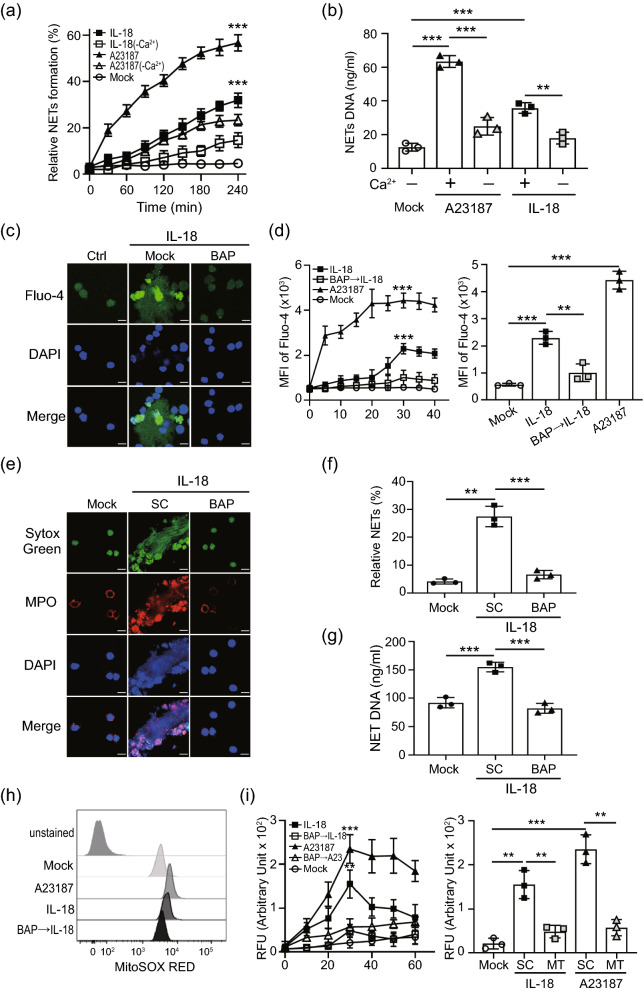
IL-18 mediated NETs formation was required calcium. (**a**, **b**) Neutrophils were stimulated with A23187 or IL-18 in the presence or absence of calcium. (**a**) The dynamic of NETs formation and (b) NETs DNA release was quantified. (**c**) dHL-60 cells were stimulated with IL-18 in the presence of intracellular calcium chelator BAPTA-AM (10 μM). The calcium mobilization in Fluo-4 AM loading neutrophils was detected by confocal microscopy. (**d**) The dynamic of calcium mobilization production in neutrophils was analyzed by using Fluo-4 AM dye with flow cytometry (left panel). At 30 min post-treatment, IL-18 induces a higher calcium mobilization compared with control cells, which was declined in the presence of calcium chelator BAPTA-AM (10 μM) (right panel). Chelation of intracellular calcium reduces IL-18 mediated- (**e**, **f**) NETs formation, (**g**) NETs DNA release, and (**h**, **i**) mitochondrial ROS production. All experiments were performed in triplicate, and data are presented as the mean ± SD. The scale bar in the IFA image represents 5 μm. *P < 0.05, **P < 0.01, ***P < 0.005. MT, MitoTEMPO; SC, solvent control.

Douda et al.^18^ demonstrated that mROS-mediated NETs formation was associated with calcium influx, we examined whether extracellular calcium was required in IL-18 induced NETs. The plate reader assays show that the levels of NETs induced by IL-18 was significantly reduced in the absence of extracellular calcium, suggesting calcium influx is crucial in IL-18 induced NETs (*P* < 0.005; Fig. 3a–b). We further confirmed the effect of calcium influx on IL-18 induced NETs by using Ca^2+^ chelator BAPTA-AM. The levels of calcium influx, NETs and mROS generation were analyzed by using confocal microscope and flow cytometry, respectively. The intracellular calcium levels were enhanced in cells treated with IL-18 (Fig. 3c–d), while it was inhibited in the presence of BAPTA-AM. Moreover, both the induced NETs formation (Fig. 3e–g) and mROS production (Fig. 3h–i) were significantly suppressed in the presence of BAPTA-AM, suggesting that IL-18-mediated NETs with mROS production might occur through enhancing calcium influx.

### Increased miR-223 levels in neutrophils of AOSD patients

**Figure 4:**
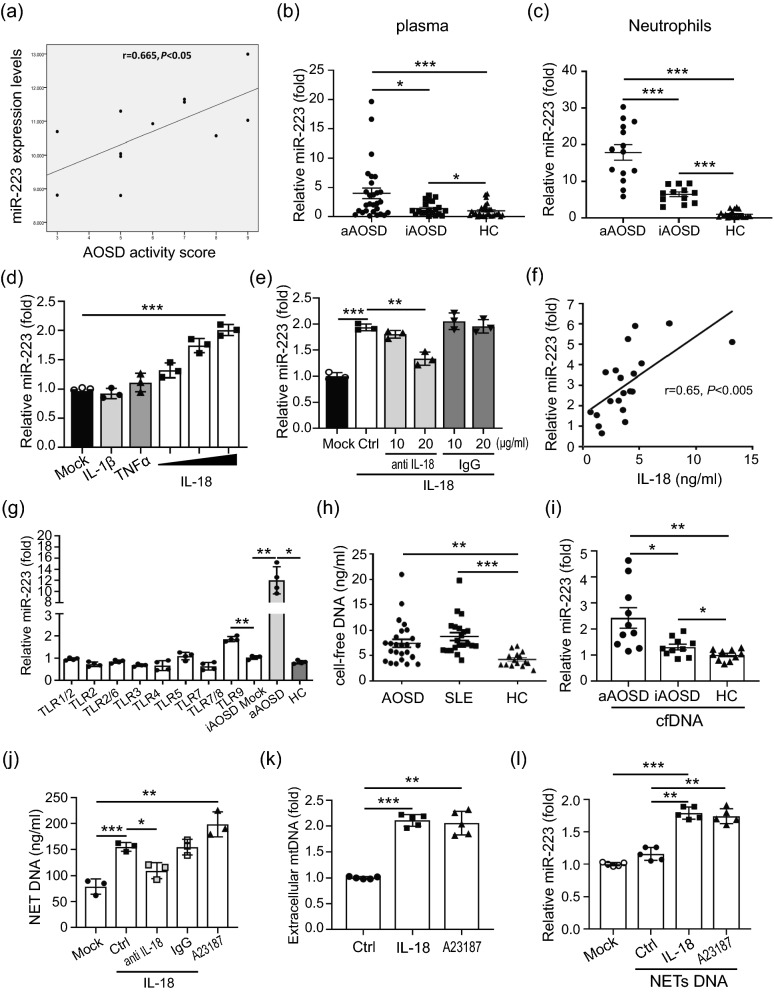
The released NETs DNA induces microRNA-223 (miR-223) upregulation through activating of Toll-like receptor 9. (**a**) Disease activity scores were positively correlated with expression levels of miR-223 in patients with AOSD. Increased miR-223 levels in (**b**) plasma and (c) neutrophils from AOSD patients. (**d**) IL-18 induced miR-223 upregulation in neutrophils is dose-dependent (the range of IL-18 concentration was from 3.125, 6.25 to 12.5 ng/ml). (**e**) IL-18 induced miR-223 upregulation was reduced specifically in the presence of anti-IL-18 antibody. (**f**) IL-18 levels were positively correlated with miR-223 expression in patients with AOSD. (**g**) The upregulated miR-223 in neutrophils from AOSD patients is due to the activation of Toll-like receptor 9. (**h**) Increased levels of cell-free DNA (cfDNA) in patients with AOSD. (**i**) Human neutrophils were treated with cfDNA from active AOSD (n = 10), inactive AOSD (n = 10) or healthy controls (n = 10), respectively. After 24 h, the intracellular miR-223 expression levels were measured by using QRT-PCR. (**j**) Anti-IL-18 antibody inhibited IL-18-induced NETs DNA release specifically. (**k**) dHL-60 cells were treated with IL-18 or A23187, and the levels of released mtDNA was measured by using QRT-PCR. (l) IL-18 induced NETs DNA contributes miR-223 upregulation. All experiments were performed in triplicate, and data are presented as the mean ± SD. *P < 0.05, **P < 0.01, ***P < 0.005.

Given the positive association of elevated miR-223 expression levels with AOSD activity scores (Fig. 4a and Supplementary Fig. S3a), we revealed higher circulating miR-223 levels in active AOSD patients (Fig. 4b) than in inactive AOSD patients or HC. Similarly, the miR-223 expression levels in neutrophils were significantly higher in active AOSD patients (Fig. 4c) compared with inactive AOSD patients or HC. A decrease of miR-223 expression was observed in active AOSD patients after therapy (Supplementary Fig. S3b).

### IL-18 induced miR-223 upregulation in neutrophils

To dissect the correlation between miR-223 and AOSD-related cytokines, we evaluated intracellular miR-223 expression in dHL-60 cells treated with IL-18, IL-1β (50 ng/ml), or TNFα (100 ng/ml). The results revealed that IL-18 could induce miR-223 expression in a dose-dependent manner (*P* < 0.005, Fig. 4d). Moreover, the IL-18 induced miR-223 upregulation is blocked in presence of anti-IL-18 antibody (*P* < 0.01, Fig. 4e), suggesting miR-223 expression is regulated by IL-18 specifically. Additionally, we observed a positive correlation between plasma IL-18 levels and miR-223 levels in AOSD patients (r = 0.65, *P* < 0.005; Fig. 4f).

### Extracellular DNA from AOSD patients induced miR-223 expression

Next, we investigated whether Toll-like receptors (TLRs) might regulate miR-223 expression. The miR-223 expression levels were significantly upregulated in AOSD patient neutrophils treated with TLR9 ligands (Fig. 4g), suggesting that double-stranded DNA/TLR9 might contribute to miR-223 expression in neutrophils from AOSD patients.

Extracellular cell-free (cf) DNA has been reported to be implicated in autoimmune diseases^19^. We observed increased levels of cfDNA in AOSD patients, compared with those in HC (*P* < 0.01, Fig. 4h). To assess whether extracellular DNA is associated with increased miR-223 expression in AOSD, human PMNs were treated with extracellular DNA from active AOSD, inactive AOSD or HC, respectively. Higher levels of miR-223 were induced in neutrophils treated with extracellular DNA from active AOSD patients, compared with those from inactive AOSD patients or HC (Fig. 4i).

### IL-18 induces mitochondrial DNA release to contribute miR-223 upregulation

Given that IL-18 could induce NETs formation (Fig. 3e), we further examined whether this effect is specific via IL-18 signaling. As shown in Fig. 4j, the IL-18 induced NETs DNA release is blocked in presence of anti-IL-18 antibody (*P* < 0.05), suggesting increased NETs DNA release is induced by IL-18 specifically. Given that IL-18 could induce mitochondria depolarization (Fig. 2c), we also found an increased mtDNA levels was released from cells with IL-18 or A23187 treatment, compared with control cells (Fig. 4k). We further investigate whether IL-18 contributes to miR-223 upregulation through inducing NETs DNA release. As shown in Fig. 4l, the intracellular miR-223 levels were upregulated after the addition of NETs DNA from IL-18-treated cells compared with those in control cells (*P* < 0.005).

### miR-223 inhibits IL-18 induced NETs through suppressing calcium influx

**Figure 5:**
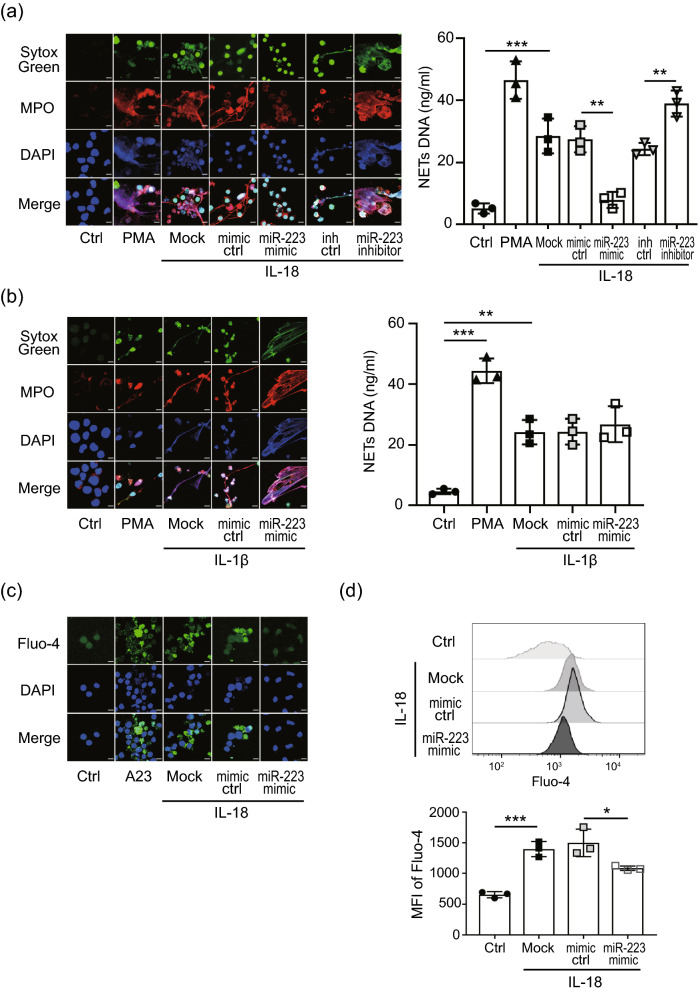
MiR-223 inhibits IL-18-induced NETs by suppressing calcium influx. (**a**) The levels of NETs in miR-223 over-expressing or miR-223 silencing cells after (**a**) IL-18 or (b) IL-1β treatment were detected and quantified. The calcium mobilization in miR-223 over-expressing cells after IL-18 treatment was detected by using Flou-4 staining and quantified with (**c**) confocal microscopy or (**d**) flow cytometry. All experiments were performed in triplicate, and data are presented as the mean ± SD. The scale bar in the IFA image represents 5 μm. **P < 0.01, ***P < 0.005.

Finally, we assessed the probable mechanisms of miR-223 on IL-18-induced NETs. Significantly lower levels of NETs formation were observed in miR-223 over-expressing cells after IL-18 induction compared with miR-223 control-expressing cells or non-transfected cells (Fig. 5a). Conversely, dramatically higher levels of NETs formation were observed in miR-223 silencing cells after IL-18 induction compared with miR-223 control-expressing cells or non-transfected cells (Supplementary Fig. S4). However, miR-223 had no significant effect on IL-1β-induced NETs (Fig. 5b). Resonate with the finding that IL-18 mediated NETs formation is mROS-dependent, ROS levels were significantly reduced in miR-223 over-expressing cells treated with IL-18 (Supplementary Fig. S4) compared with control cells, indicating an inhibitory effect of miR-223 on ROS generation.

To determine whether miR-223 suppresses IL-18-induced ROS production by inhibiting calcium influx, miR-223 over-expressing cells were activated with IL-18 and calcium influx were measured. The results indicated that miR-223 could inhibit IL-18-mediated calcium influx, as revealed by Flou-4 staining with confocal microscope, compared with those in control-expressing cells or non-transfected cells (Fig. 5c). The similar quantified result was obtained by using flow cytometry assay (Fig. 5d).

### Neutrophil–derived exo-miR-223 contributes to the inhibition of IL-18 production in macrophages via targeting NLRP3

**Figure 6:**
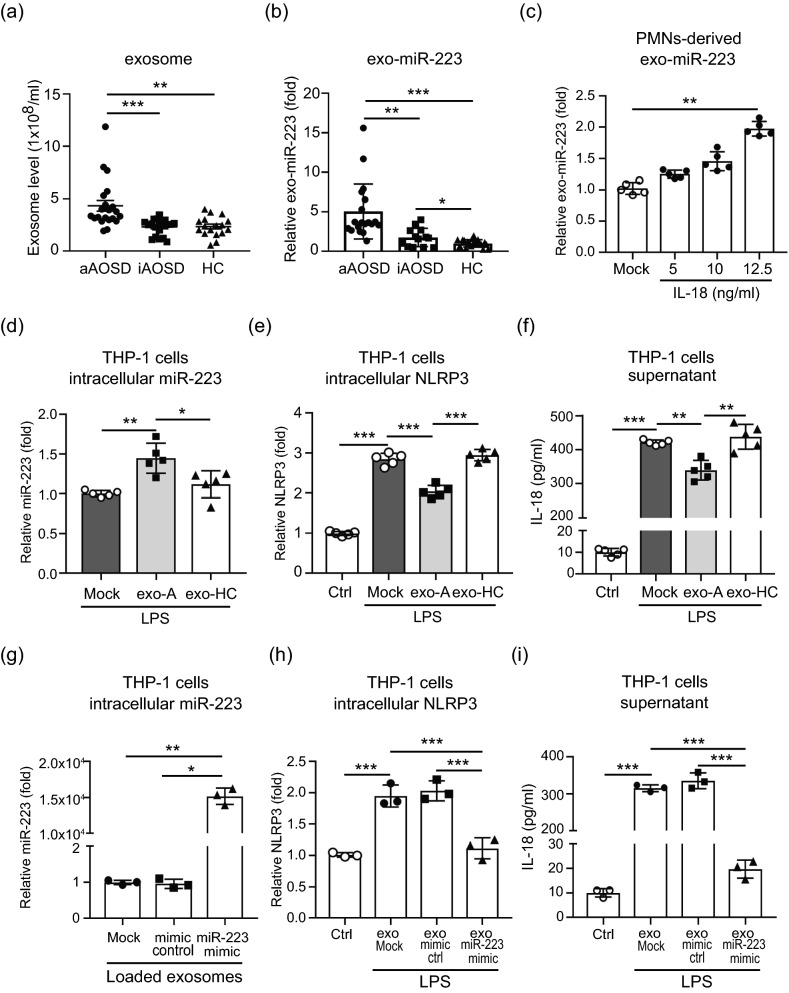
Neutrophil-derived exo-miR-223 contributes to inhibition of IL-18 secretion from macrophages by targeting NLRP3. Comparison of (a) serum-derived small extracellular vesicles (sEVs) levels, and (b) exo-miR-223 in patients with active AOSD (aAOSD), inactive AOSD (iAOSD), and healthy controls (HC). (c) IL-18 induced human neutrophil-derived exo-miR-223 levels in a dose-dependent manner. (**d**–**f**) THP-1–derived macrophages were pretreated with LPS (1 μg/ml) for 1 h to activate NLRP3, then co-cultured with the purified sEVs from active AOSD patients or HC for 24 h, respectively. The levels of (**d**) intracellular miR-223, (e) intracellular NLRP3, and (f) secretory IL-18, were measured by using QRT-PCR, and ELISA, respectively. (**g**–**i**) MicroRNA-223 in neutrophil-derived sEVs contribute to the inhibition of IL-18 secretion in macrophages. dHL-60 cell-derived sEVs were loaded with mimic miR-223, or control. THP-1–derived macrophages were pretreated with LPS (1 μg/ml) for 1 h to activate NLRP3, then co-cultured with the indicated loaded sEVs for 24 h, respectively. The levels of (**g**) intracellular miR-223, (**h**) intracellular NLRP3, and (**i**) secretory IL-18 were measured by using QRT-PCR, and ELISA, respectively. All experiments were performed in triplicate, and data are presented as the mean ± SD. The scale bar in the IFA image represents 5 μm. **P < 0.01, ***P < 0.005.

Recent studies have demonstrated that sEVs can carry miRNAs and play a key role in intercellular communications^20–23^. We isolated EVs from sera of AOSD patients or HC. To validate the purity of EVs used in this study, we measured particle size of EVs (Zetasizer Nano ZS90, Malvern Instruments, Malvern, UK), and detected EVs-specific (Alix, TSG101, CD63, CD9, and CD81) surface marker using immunoblotting. The result proving the average particle size of EVs we extracted is 90.80 nm and the particle size of major peak is 147.8 nm (81.3% intensity), both consistent with the size definition of typical sEVs (< 100 nm or < 200 nm)^24^. (Supplementary Fig. S5a). Moreover, these sEVs expressed specific EVs markers (Supplementary Fig. S5b). These results indicate that the sera-derived EVs we extracted is majorly composed by sEVs. Moreover, most sEVs of AOSD patients expressed neutrophil-specific surface marker (CD66b), suggesting they were neutrophil-derived. Increased levels of neutrophil-derived sEVs (Fig. 6a) and exo-miR-223 (Fig. 6b) were observed in patients with active AOSD, as compared with inactive AOSD patients or HC. Additionally, we observed that IL-18 is not only able to induce the upregulation of intracellular miR-223 (Fig. 4d), but can also increase human neutrophil-derived exo-miR-223 levels (Fig. 6c). Given that miR-223 can inhibit IL-18 expression in macrophages by targeting NLR family pyrin domain containing 3 (NLRP3)^23^, we hypothesized neutrophil-derived miR-223 may feedback and suppress IL-18 production in macrophages through sEVs transmission to inhibit IL-18-mediated NETs. To examine the effect of sEVs from AOSD patients on NLRP3 expression, THP-1-derived macrophages were pretreated with lipopolysaccharides (LPS, 1 μg/ml) for 1 h to activate NLRP3, then co-cultured with the purified sEVs from active AOSD patients or HC subjects for 24 h, respectively. An increased intracellular miR-223 level was detected in macrophages with the addition of AOSD patient-derived sEVs (Fig. 6d), compared with HC-derived sEVs, or non-treatment control. A greater expression of NLRP3 was detected in cells with LPS induction (Fig. 6e, Supplementary Fig. S6a). The LPS-induced NLRP3 expression was suppressed after adding sEVs from AOSD patients, but no significant difference was revealed in cells treated with sEVs from HC subjects. Similarly, dramatically increased IL-18 levels were induced in cells with LPS stimulation, then suppressed significantly after adding sEVs from AOSD patients (Fig. 6f). These results suggested that AOSD patient-derived sEVs may inhibit IL-18 production in macrophages by targeting NLRP3 through exo-miR-223 transmission.

To support our findings, we loaded miR-223 mimic or control into dHL-60 cell-derived sEVs using electroporation in accordance with our previous published report^21^. To validate that sEVs were neutrophil in origin, we detected EVs- (Alix, or TSG101) and neutrophil-specific (CD66b) surface markers using immunoblotting (Supplementary Fig. S5c). The levels of encapsulated miR-223 were measured using QRT-PCR. Approximately, a 1.64-fold increased expression of miR-223 was observed in dHL-60 cell-derived sEVs after electroporation with miR-223 mimic, indicating its effective loading by electroporation (Supplementary Fig. S5d). The sEVs were then added to THP-1-derived macrophages with LPS induction. After 48 h, an increased intracellular miR-223 level (Fig. 6g, *P* < 0.01) and decreased NLRP3 expression (Fig. 6h, Supplementary Fig. S6b) was observed in cells with the addition of mimic miR-223-loaded sEVs. Furthermore, the IL-18 levels were significantly decreased in cells that were treated with mimic miR-223-loaded sEVs compared with those with the addition of control-loaded sEVs or non-loaded sEVs (Fig. 6i, P < 0.005). Conversely, a decreased intracellular miR-223 level was detected in macrophages that were treated with miR-223 inhibitor loaded sEVs compared with those with the addition of control-loaded sEVs or non-loaded sEVs (*P* < 0.005, Supplementary Fig. S7a). An increased NLRP3 expression (Supplementary Fig. S7b) and elevated secretory IL-18 levels (Supplementary Fig. S7c) were measured in miR-223 silencing cells.

## Discussion

IL-18, a crucial cytokine of AOSD^25–28^, plays an important role in inflammation^29^. Herein, we demonstrated that IL-18 induced NETs via enhancing calcium influx to neutrophils. The increased calcium influx promoted Akt activation, which may have participated in mROS production with the release of NETs enriched in oxidized mtDNA. The extracellular NETs DNA upregulated miR-223 expression through activating TLR9. The miR-223 was able to inhibit IL-18-mediated NETs via suppressing calcium influx. Additionally, neutrophil–derived sEVs carried miR-223 could feed back and suppress the NLRP3 inflammasome and IL-18 production in macrophages. These findings suggest that a fine-tuned mechanism between inflammatory (IL-18 induced NETs) and anti-inflammatory (miR-223) factors is important in the pathogenesis of AOSD.

We observed that both 8-OHdG levels and spontaneous citrullinated histone expression are higher in active AOSD patients than in HCs [7, unpublished data]. Calcium influx plays a key role in the formation of NETs by being implicated in ROS generation and histone citrullination^18,30^. Overloading mitochondrial matrix Ca^2+^ enhances the production of ROS, which induce the damage of the permeability transition pore, leading to apoptosis^30^. Zhou et al*.*^31^ revealed that IL-18 significantly induces calcium influx in human umbilical vein endothelial cells. We demonstrated that IL-18 mediated NETs through increasing calcium influx to neutrophils, thus, damaging the mitochondrial membrane potential and inducing mROS production. Additionally, IL-18 induced the production of citrullinated histones in neutrophils.

The NETs of patients with autoimmune diseases have been shown to contain oxidized mtDNA and stimulate type I interferon signaling^17^. Nakahira et al*.*^32^ showed that mROS and mtDNA contribute to macrophage NLRP3 and AIM2 inflammasome activation. Shimada et al*.*^33^ further demonstrated that oxidized mtDNA activates the NLRP3 inflammasome during apoptosis. Herein, we showed that IL-18 induced mtDNA release from neutrophils, resulting increased levels of NETs enriched in oxidized mtDNA in plasma from AOSD patients. We further demonstrated that miR-223 expression was upregulated in neutrophils after stimulation with extracellular DNA from active AOSD patients, or IL-18-treated cells.

MiRNAs, small regulatory RNAs, have a central role in the regulation of gene expression and serve as biomarkers for various pathological conditions^34^. We revealed that increased circulating miR-223 levels were positively correlated with disease activity scores and IL-18 levels in AOSD patients. MiR-223 shows specific expression in the hematopoietic system^35^. One study has demonstrated that mtDNA/TLR9 activates a negative feedback pathway through the induction of miR-223 to limit neutrophil over-activation and liver injury^36^. Our results revealed that NETs DNA isolated from IL-18-treated cells upregulate miR-223 expression on neutrophils via TLR9 activation. Moreover, we revealed that miR-223 could inhibit IL-18-induced NETs and attenuate ROS generation in dHL-60 cells. Our results were similar to those reported by Li et al., showing that deletion of the miR-223 gene exacerbates hepatic neutrophil infiltration and ROS production^37^. An in-depth study is needed to confirm our hypothesis.

Kanno et al.^38^ showed that IL-18 could stimulate the release of glutamate. Our metabolomic analysis showed an increased level of pyroglutamic acid in AOSD patients compared to healthy controls, suggesting glutamate may be involved in the pathogenic of this disease^39^. A study reported that a glutamate receptor blocker inhibits NETs by reducing glutamate release, and attenuating calcium influx as well as ROS production^40^. Harraz et al.^41^ demonstrated that miR-223 inhibits calcium influx by targeting glutamate receptor. We showed that miR-223 was able to suppress IL-18-mediated NETs by decreasing intracellular calcium. However, the detailed actions of miR-223 in calcium influx and glutamate release in AOSD pathogenesis require further study in the future.

In addition to regulation of calcium influx in neutrophils, we demonstrated that sEVs carry neutrophil-derived miR-223 could repress IL-18 production in macrophages by targeting NLRP3, and thus suppress IL-18-mediated NETs formation. Besides, significantly increased IL-18 expression levels were detected in THP-1 cell treated with miR-223 silencing compared to control cells or without transfection cells, consistent with the results of the previous reports^23,42^. Although increased levels of miR-223 were revealed in plasma (Fig. 4b), neutrophils (Fig. 4c), and sera-derived exo-miR-223 (Fig. 6b) of active AOSD patients, we observed a lower level of miR-223 in PBMCs from active AOSD patients compared to inactive AOSD patients (Supplementary Fig. S2b). In addition, higher levels of serum IL-18 were observed in our active AOSD patients compared to inactive AOSD patients (Supplementary Fig. S2a). These results suggested that miR-223 could inhibit IL-18 expression and an impaired expression of miR-223 may upregulate IL-18 expression as shown in PBMCs from active AOSD patients. Therefore, we hypothesized that an impaired uptake of exo-miR-223 may exist in macrophages (IL-18 producing cell), which play a crucial role in AOSD pathogenesis. Further in-depth studies need to confirm our hypothesis. Besides, NETs formation was increased in miR-223 silencing cells after stimulating IL-18 compared to miR-223-control cells (Fig. 5a). These observations suggest that AOSD patients with lower expression levels of miR-223 in PBMCs may have a higher disease activity. Further large studies are required to confirm our data herein.

Extracellular vesicles play crucial roles in intercellular communication and regulation of cell signaling^11^. Neutrophil-derived sEVs can regulate adaptive immune responses by affecting other types of cells^43^. Genschmer et al*.*^44^ demonstrated that activated neutrophil-derived sEVs degrade the extracellular matrix by harboring neutrophil elastase, causing the hallmarks of chronic obstructive pulmonary disease. Increasing evidence has indicated that neutrophil-derived sEVs carry specific miRNAs that can be functionally transferred to recipient cells and are, therefore, involved in immune modulation^12,45,46^. In the present study, we showed IL-18 induced neutrophil activation, followed by intracellular miR-223 upregulation and exo-miR-223 elevation. Neutrophil-derived exo-miR-223 was able to feed back and control IL-18 secretion from macrophages, resulting in repression of NETs. In addition, we showed that miR-223-loaded sEVs were able to suppress NLRP3 inflammasome expression and decrease IL-18 secretion from macrophages. Given a pathogenic role of IL-18 in AOSD, blocking IL-18 with recombinant IL18 BP (tadekinig alfa) has therapeutic efficacy for AOSD patients in a phase II clinical trial^47^. Although IL-18-targeted biologic therapy may be effective in AOSD patients, the economic burden and potential risk of severe infection should be considered. Therefore, there is an unmet need to develop therapeutic agents with novel mechanisms of action. We revealed that miR-223 could inhibit IL-18-mediated NETs formation through blocking calcium influx, suggesting that the calcium channel blockers might be potential therapeutic agents. Based on the findings that miR-223 could directly target NLRP3^42,48^, and suppress NLRP3 inflammasome and IL-18 production in our study, miR-223-based therapeutic may hold promise as future novel therapeutics for AOSD. In addition, EVs have been considered as potential vector for therapeutic applications^49^, and our result suggests that miR-223-loaded sEVs might be potential therapeutic agents for inflammatory disease therapies. Further in vivo studies are required to confirm our observations.

Despite the novel findings presented in this pilot study, there are some limitations. Given that AOSD is a clinically heterogeneous and rare disease^50^, the number of active patients enrolled in our study was quite limited. Besides IL-18, other inflammatory cytokines, or other components in the serum of AOSD patients may be involved in the formation of NETs, so more in-depth studies are required to dissect their roles and biological functions. Additionally, this was a cross-sectional study; thus, the possibility that miRNA expression changed with therapeutic strategies cannot be excluded.

**Figure 7:**
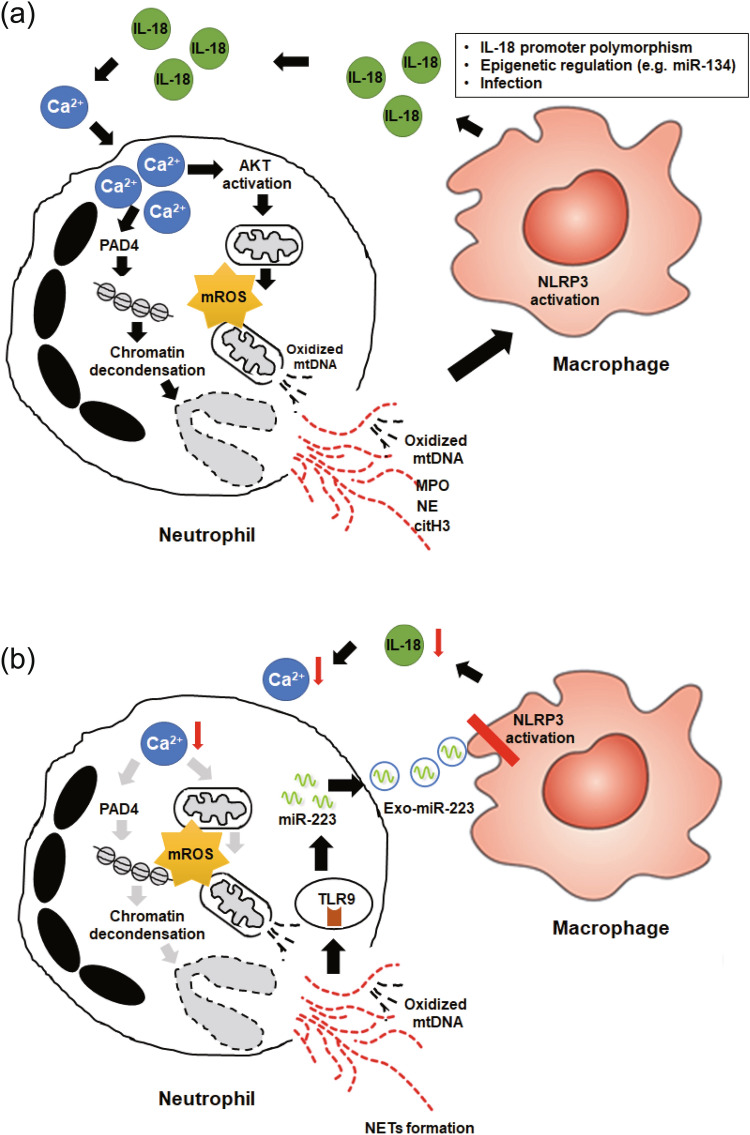
Proposed model for the biological role of IL-18 mediated NETs and miR-223 in pathogenesis of AOSD based on the results of this study. (**a**) IL-18 induces NETs by enhancing calcium influx in neutrophils. IL-18 induced calcium influx promotes Akt activation, which may participate in mROS production and then enhance oxidized mtDNA release. (**b**) The NETs enriched in oxidized mtDNA induces miR-223 upregulation through activating TLR9. Neutrophil-derived small extracellular vesicles (sEVs) carried miR-223, which could repress IL-18 production in macrophages by targeting NLRP3, thus decrease IL-18 induced calcium influx into neutrophils and inhibit IL-18 mediated NETs formation.

In summary, we have revealed that IL-18 induces NETs by enhancing calcium influx into neutrophils. The IL-18-induced calcium influx may promote Akt activation, which participates in mROS production with an increased release of oxidized mtDNA (Fig. 7a). The NETs enriched in oxidized mtDNA may then upregulate miR-223 expression by activating TLR9. To confront NETs-related inflammation, an elevated expression of miR-223 could inhibit IL-18 mediated NETs by suppressing the calcium influx into neutrophils, and repressing IL-18 expression in macrophages through sEVs transmission (Fig. 7b). Our results suggest that calcium channel blockers, mROS inhibitors, and miR-223 may be potential therapeutic agents for autoinflammatory diseases such as AOSD.

AOSD is a systemic inflammatory disorder with unknown etiology, and an important cause of unknown fever in adults. IL-18 is a key factor of AOSD and plays an important role in inflammation. Increasing evidence reveals that an elevated NETs was observed in AOSD patients, but the regulatory mechanism is unclear. Here we demonstrated a novel relationship between IL-18 and production of NETs, which promotes inflammatory disorder in AOSD. Moreover, we demonstrated that increased NETs upregulate miR-223 expression by activating TLR9. Upregulation of miR-223 could inhibit IL-18 mediated NETs by suppressing the calcium influx into neutrophils, and repressing IL-18 expression in macrophages through sEVs transmission. MiR-223, IL-18 and NETs may become potential diagnostic biomarkers or therapeutic targets for AOSD.

## Methods

### Subjects

In this prospective study, 38 patients with active AOSD were consecutively enrolled, each fulfilling the Yamaguchi criteria^51^. Patients with infections, malignancies, or other rheumatic diseases were excluded. Disease activity was assessed using a modified Pouchot score described by Rau et al.^52^, with active AOSD defined as having an activity score of 4 or higher^53^. All patients received corticosteroids and/or non-steroidal anti-inflammatory drugs (NSAIDs) at an active status. Defined as in previous studies^54^, all the enrolled AOSD patients who were followed for at least one year were classified into two subtypes of disease course: a systemic subtype that includes monocyclic and polycyclic form, and the other chronic articular subtype. Twenty-six healthy volunteers were enrolled as control subjects. The Institutional Review Board of China Medical University Hospital Research Ethics Committee approved this study (CMUH107-REC3-014) and the written informed consent of all participants was obtained according to the Declaration of Helsinki.

### Cell culture

The polymorphonuclear neutrophils (PMNs) were immediately isolated from venous blood using Polymorphprep (AXIS-SHIELD, Dundee, UK) density gradient centrifugation. The PMNs or human promyelocytic leukemia cell line HL-60 cells (ATCC CCL-240) were grown in RPMI medium 1640 (Thermo Fisher Scientific, Fremont, CA, USA) supplemented with 10% FBS, 1 × nonessential amino acids, 100 units/ml penicillin, 100 units/ml streptomycin, and 2% autologous serum (for PMNs culture only) in an incubator containing 5% CO_2_ at 37 °C. To induce differentiation into neutrophil-like cells (dHL-60), HL-60 cells were grown in media and treated with 1.3% dimethyl sulfoxide (Sigma-Aldrich, St Louis, MO, USA) for 72 h.

### Immunofluorescence assay (IFA)

Human PMNs or dHL-60 cells were incubated in fibronectin (200 μg/ml)-coated culture slides in the presence of the indicated inhibitors: diphenyleneiodonium (DPI, 25 μM), MitoTEMPO (10 μM) for 1 h and activated with phorbol myristate acetate (PMA, 25 nM), A23187 calcium ionophore (25 μM), IL-1β (50 ng/ml), or IL-18 (12.5 ng/ml) for 4 h at 37 °C, fixed with 4% paraformaldehyde, permeabilized with PBS containing 1% BSA and 0.2% saponin and then blocked for 1 h in PBS containing 2% BSA. After that, cells were incubated with mouse myeloperoxidase (MPO) antibody (Santa Cruz Biotechnology, Dallas, TX, USA) followed by a secondary antibody (Thermo Fisher Scientific) for MPO detection. The DNA were stained with 5 μM Sytox Green (Thermo Fisher Scientific) for 10 min after the fixation and permeabilization. Coverslips were mounted onto glass slides with DAPI-containing Slow-Fade mounting medium (Thermo Fisher Scientific), and images were observed and recorded on an Olympus FV1000 laser scanning confocal microscope. Images were analyzed by using FV10-ASW version 4.2 software.

### Quantification of NETs

The levels of NETs formation were quantified by measuring Sytox Green (Thermo Fisher Scientific) intensity by plate reader (Gemini EM, Molecular Devices, Sunnyvale, CA, USA)^18^. In brief, cells were seeded at 5 × 10^4^ cells per well in a 96-well plate in the culture media in the presence of Sytox Green. Fluorescence was measured at 30 min intervals for 240 min after the activation of cells. The fluorescence readout obtained from cells lysed with 0.5% (vol/vol) Triton X-100 (Sigma-Aldrich) was considered as 100% DNA release, and the relative NETs formation of individual treatment was calculated at each time point^18^. NETs DNA release in the supernatant of stimulated cells was detected by using Quant-iT PicoGreen dsDNA Assay kit (Thermo Fisher Scientific) according to the manufacturer’s instructions.

### Quantification of ROS production and mitochondrial membrane potential

The levels of cytosolic/mitochondrial ROS (mROS) were analyzed by using fluorescent dye 2′,7′–dichlorofluorescin diacetate (DCFDA) (Abcam, Cambridge, MA, USA), dihydrorhodamine (DHR) 123 (Thermo Fisher Scientific), or MitoSOX Red (Thermo Fisher Scientific) staining and quantified by flow cytometry (FACSCanto II, BD Biosciences, San Jose, CA, USA) or plate reader. Data of flow cytometry were analyzed by the CellQuest software and expressed as the mean fluorescence intensity (MFI) of cytosolic/mitochondrial ROS.

The mitochondrial membrane potential was detected by using JC1 staining (Abcam) and quantified with a plate reader.

### Quantification of intracellular calcium level

The intracellular calcium concentration was assessed by using Fluo-4 AM calcium indicator dye (Thermo Fisher Scientific) combined with confocal microscope, and quantified by using flow cytometry. Briefly, human neutrophils or dHL-60 cells were incubated in HBSS-Mg^2+^ (calcium free) media with 4 µM Fluo-4-AM for 15 min at 37 °C. After washing, the cells were resuspended in RPMI and stimulated with either calcium ionophore A23187 or IL-18 in the presence or absence of calcium chelator BAPTA-AM (1,2-Bis(2-aminophenoxy) ethane-N,N,N′,N′-tetra acetic acid acetoxymethyl ester, 10 µM, Thermo Fisher Scientific), and Fluo-4-AM fluorescence intensities was measured at 5 min intervals for 40 min after the activation of cells by using flow cytometry.

### MicroRNA isolation and quantification

Total RNAs were extracted using TRIzol Reagent (Thermo Fisher Scientific) and purified using a RNeasy MinElute Cleanup kit (QIAGEN, Valencia, CA, USA), according to the manufacturer’s instructions. Purified RNAs were quantified at OD260 and 280 nm using a NanoDrop spectrophotometer (Thermo Fisher Scientific).

MicroRNA expression was quantified using a TaqMan MicroRNA Assay kit (Thermo Fisher Scientific), according to the manufacturer’s protocol. Quantitative reverse transcription PCR (QRT-PCR) reactions were performed on the StepOnePlus Real-Time PCR System (Thermo Fisher Scientific), using a standard protocol.

### Extracellular mitochondrial DNA quantification

Cell-free DNA (cfDNA) from the plasma or supernatants of IL-18/A23187-stimulated neutrophils were extracted by using the QIAamp DNA Mini kit (QIAGEN), and the concentration of the purified cfDNA was qualified using a NanoDrop spectrophotometer (Thermo Fisher Scientific). Extracellular mitochondrial DNA (mtDNA) was amplified by using QRT-PCR with the human mtDNA-specific primers (mtF3212: 5′-CACCCAAGAACAGGGTTTGT-3′ and mtR3319: 5′-TGGCCATGGGATTGTTGTTAA-3′) and LightCycler FastStart DNA Master SYBR Green I (Roche Applied Science, Mannheim, Germany). For extracellular mtDNA quantification, results were shown as a ratio of mtDNA to 18S nuclear DNA.

### Immunoblotting

The cells with different treatments were lysed in RIPA buffer (25 mM Tris–HCl pH 7.6, 150 mM NaCl, 1% NP-40, 1% sodium deoxycholate, and 0.1% SDS) containing a protease inhibitor cocktail (Roche Applied Science). Twenty micrograms of total protein from sEVs lysate were loaded and separated on a standard sodium dodecyl sulfate (SDS)-polyacrylamide gel electrophoresis (PAGE) gel and transferred to a polyvinylidene difluoride (PVDF) membrane (Millipore, Billerica, MA, USA). The membranes were incubated with primary antibodies to the NETs signaling molecules, followed by peroxidase-conjugated secondary antibodies. The results were detected using a charge-coupled device (CCD) camera-based imager (GE Healthcare Life Sciences, Chicago, IL, USA) after membrane incubation with enhanced chemiluminescence (ECL) substrates (Millipore). The protein levels of NETs signaling molecules were normalized to β-actin.

### Extracellular vesicles isolation and quantification

Samples were centrifuged at 2500 rpm for 10 min at 4 °C to remove cell debris, then filtered through a 0.22 μm filter. The serum- and dHL-60 cell–derived EVs were exacted by ExoQuick exosomes precipitation solution (System Biosciences, Palo Alto, CA, USA) according to the manufacturer’s instructions. The purified sEVs were confirmed using immunoblotting. The sEVs were quantified using a direct ELISA-based method to quantify the EVs surface marker CD63 according to the manufacturer’s instructions (System Biosciences).

### Loading of miR-223 mimic, or control into sEVs

Electroporation was used in loading miR-223 mimic, or control into dHL-60 cells–derived sEVs. In brief, 0.1 µmole of miR-223 mimic, or control (Thermo Fisher Scientific, USA) were added to 20 µl of dHL-60 cells–derived sEVs sample (approximately 5 × 10^8^ particles). The mixtures were electroporated at 500 pulse voltage/10 pulse width (ms)/ 3 pulse number using a Neon Transfection system (Thermo Fisher Scientific). After electroporation, the mixture was immediately treated with one unit of RNase A (QIAGEN) for 30 min, followed by the addition of the 2 µl RNase inhibitor. MiR-223 mimic-, or control-loaded sEVs were extracted using ExoQuick exosomes precipitation solution (System Biosciences) according to the manufacturer’s instructions.

### Statistical analysis

An unpaired, two-tailed Student’s *t* test was used for between-group comparisons. A one-way analysis of variance (ANOVA) with the post hoc Bonferroni test was used for multiple comparisons. The correlation coefficient was calculated using Spearman’s correlation test. *P* values < 0.05 were statistically significant and tests were performed by using GraphPad Prism version 8.0.0 (GraphPad Software, San Diego, CA, USA).

## Supplementary Information

Supplementary Information.

## References

[1] Nathan C (2006). Neutrophils and immunity: Challenges and opportunities. Nat. Rev. Immunol..

[2] Mantovani A, Cassatella MA, Costantini C, Jaillon S (2011). Neutrophils in the activation and regulation of innate and adaptive immunity. Nat. Rev. Immunol..

[3] Jorch SK, Kubes P (2017). An emerging role for neutrophil extracellular traps in noninfectious disease. Nat. Med..

[4] Ruscitti P, Giacomelli R (2018). Pathogenesis of adult onset Still’s disease: Current understanding and new insights. Expert. Rev. Clin. Immunol..

[5] Hu Q (2019). Increased neutrophil extracellular traps activate NLRP3 and inflammatory macrophages in adult-onset Still's disease. Arthritis Res. Ther..

[6] Torres-Ruiz J (2019). The role of low density granulocytes and NETosis in the pathogenesis of adult-onset Still’s disease. Clin. Exp. Rheumatol..

[7] Bartel DP (2004). MicroRNAs: Genomics, biogenesis, mechanism, and function. Cell.

[8] Lodish HF, Zhou B, Liu G, Chen CZ (2008). Micromanagement of the immune system by microRNAs. Nat. Rev. Immunol..

[9] Liao TL (2017). Upregulation of circulating microRNA-134 in adult-onset Still’s disease and its use as potential biomarker. Sci. Rep..

[10] Johnnidis JB (2008). Regulation of progenitor cell proliferation and granulocyte function by microRNA-223. Nature.

[11] Simons M, Raposo G (2009). Exosomes—vesicular carriers for intercellular communication. Curr. Opin. Cell Biol..

[12] Shao S (2019). Neutrophil exosomes enhance the skin autoinflammation in generalized pustular psoriasis via activating keratinocytes. FASEB J..

[13] Butin-Israeli V (2016). Deposition of microparticles by neutrophils onto inflamed epithelium: A new mechanism to disrupt epithelial intercellular adhesions and promote transepithelial migration. FASEB J..

[14] Lood C (2016). Neutrophil extracellular traps enriched in oxidized mitochondrial DNA are interferogenic and contribute to lupus-like disease. Nat. Med..

[15] Choi JH (2003). Serum cytokine profiles in patients with adult onset Still’s disease. J. Rheumatol..

[16] Chen DY, Lan JL, Lin FJ, Hsieh TY, Wen MC (2004). Predominance of Th1 cytokine in peripheral blood and pathological tissues of patients with active untreated AOSD. Ann. Rheum. Dis..

[17] Neeli I, Khan SN, Radic M (2008). Histone deimination as a response to inflammatory stimuli in neutrophils. J. Immunol..

[18] Douda DN, Khan MA, Grasemann H, Palaniyar N (2015). SK3 channel and mitochondrial ROS mediate NADPH oxidase-independent NETosis induced by calcium influx. Proc. Natl. Acad. Sci. U. S. A..

[19] Duvvuri B, Lood C (2019). Cell-Free DNA as a biomarker in autoimmune rheumatic diseases. Front. Immunol..

[20] Li D (2016). Osteoclast-derived exosomal miR-214-3p inhibits osteoblastic bone formation. Nat. Commun..

[21] Liao TL (2018). Rituximab may cause increased hepatitis C virus viremia in rheumatoid arthritis patients through declining exosomal MicroRNA-155. Arthritis Rheumatol..

[22] Pan Y (2019). Adipocyte-secreted exosomal microRNA-34a inhibits M2 macrophage polarization to promote obesity-induced adipose inflammation. J. Clin. Invest..

[23] Haneklaus M (2012). Cutting Edge: miR-223 and EBV miR-BART15 Regulate the NLRP3 Inflammasome and IL-1β Production. J. Immunol..

[24] Théry C (2018). Minimal information for studies of extracellular vesicles 2018 (MISEV2018): A position statement of the International Society for Extracellular Vesicles and update of the MISEV2014 guidelines. J. Extracell. Vesicles..

[25] Sugiura T (2002). Association between adult-onset Still's disease and interleukin-18 gene polymorphisms. Genes Immunol..

[26] Jung KH (2014). Interleukin-18 as an efficient marker for remission and follow-up in patients with inactive adult-onset Still's disease. Scand. J. Rheumatol..

[27] Girard C (2016). Elevated serum levels of free interleukin-18 in adult-onset Still's disease. Rheumatology (Oxford).

[28] Hsieh CW (2017). Elevated expression of the NLRP3 inflammasome and its correlation with disease activity in adult-onset still disease. J. Rheumatol..

[29] Leung BP (2001). A role for IL-18 in neutrophil activation. J. Immunol..

[30] Hann J, Bueb JL, Tolle F, Brechard S (2020). Calcium signaling and regulation of neutrophil functions: Still a long way to go. J. Leukoc. Biol..

[31] Zhou G (2009). IL-18 accelerates the cell apoptosis by up-regulating cysteinyl leukotriene 2 receptor expression in human umbilical vein endothelial cells at the early stage of administration. Vascul. Pharmacol..

[32] Nakahira K (2011). Autophagy proteins regulate innate immune responses by inhibiting the release of mitochondrial DNA mediated by the NALP3 inflammasome. Nat. Immunol..

[33] Shimada K (2012). Oxidized mitochondrial DNA activates the NLRP3 inflammasome during apoptosis. Immunity.

[34] Chua JH, Armugam A, Jeyaseelan K (2009). MicroRNAs: Biogenesis, function and applications. Curr. Opin. Mol. Ther..

[35] Chen CZ, Li L, Lodish HF, Bartel DP (2004). MicroRNAs modulate hematopoietic lineage differentiation. Science.

[36] He Y (2017). Hepatic mitochondrial DNA/Toll-like receptor 9/MicroRNA-223 forms a negative feedback loop to limit neutrophil overactivation and acetaminophen hepatotoxicity in mice. Hepatology.

[37] Li M (2017). MicroRNA-223 ameliorates alcoholic liver injury by inhibiting the IL-6-p47^phox^-oxidative stress pathway in neutrophils. Gut.

[38] Kanno T, Nagata T, Yamamoto S, Okamura H, Nishizaki T (2004). Interleukin-18 stimulates synaptically released glutamate and enhances postsynaptic AMPA receptor responses in the CA1 region of mouse hippocampal slices. Brain Res..

[39] Chen, D. Y. *et al. *Metabolic disturbances in adult-onset Still's disease evaluated using liquid chromatography/mass spectrometry-based metabolomic analysis. *PLoS One. ***11**, e0168147 (2016). 10.1371/journal.pone.0168147. 10.1371/journal.pone.0168147 PMC5179000 28005947

[40] Del Arroyo AG (2019). NMDA receptor modulation of glutamate release in activated neutrophils. EBioMedicine.

[41] Harraz MM, Eacker SM, Wang X, Dawson TM, Dawson VL (2012). MicroRNA-223 is neuroprotective by targeting glutamate receptors. Proc Natl Acadf Sci U S A..

[42] Bauernfeind F, Rieger A, Schildberg FA, Knolle PA, Schmid-Burgk JL, Hornung V (2012). NLRP3 inflammasome activity is negatively controlled by miR-223. J. Immunol..

[43] Fernandez-Messina L (2015). Immunomodulatory role of microRNAs transferred by extracellular vesicles. Biol Cell..

[44] Genschmer KR (2019). Activated PMN exosomes: Pathogenic entities causing matrix destruction and disease in the lung. Cell.

[45] Salvi, V. V. *et al*. Exosome-delivered microRNAs promote IFN-alpha secretion by human plasmacytoid DCs via TLR7. *JCI Insight. ***3**, e98204 (2018). 10.1172/jci.insight.98204 10.1172/jci.insight.98204 PMC6012509 29769437

[46] Eken C (2008). Polymorphonuclear neutrophil-derived ectosomes interfere with the maturation of monocyte-derived dendritic cells. J. Immunol..

[47] Gabay C (2018). Open-label, multicentre, dose-escalating, phase II clinical trial on the safety and efficacy of tadekining alfa (IL-18BP) in adult-onset Still’s disease. Ann. Rheum. Dis..

[48] Boxberger N, Hecker M, Zettl UK (2019). Dysregulation of inflammasome priming and activation by microRNAs in human immune-mediated diseases. J. Immunol..

[49] Brook AC (2019). Neutrophil-derived miR-223 as local biomarker of bacterial peritonitis. Sci. Rep..

[50] Magadur-Joly G (1995). Epidemiology of adult Still’s disease: Estimate of the incidence by a retrospective study in west France. Ann. Rheum. Dis..

[51] Yamaguchi M (1992). Preliminary criteria for classification of adult Still's disease. J. Rheumatol..

[52] Rau M (2010). Clinical manifestations but not cytokine profiles differentiate adult-onset Still’s disease and sepsis. J. Rheumatol..

[53] Ahn MH (2019). Neutrophil extracellular traps may contribute to the pathogenesis in adult-onset Still’s disease. J. Rheumatol..

[54] Kirchner T (2012). The impact of various reactive oxygen species on the formation of neutrophil extracellular traps. Mediators Inflamm.

