# Supplementary material for: MicroRNA-223 inhibits neutrophil extracellular traps formation through regulating calcium influx and small extracellular vesicles transmission
Source: Sci Rep. 2021 Aug 3;11:15676. doi: 10.1038/s41598-021-95028-0 (PMC8333426; doi:10.1038/s41598-021-95028-0)

Supplementary information

MicroRNA-223 inhibits neutrophil extracellular traps formation through regulating calcium influx and small extracellular vesicles transmission

Tsai-Ling Liao[1,2,3], Yi-Ming Chen[1,4,§], Kuo-Tung Tang[4,§], Po-Ku Chen[5,6,7], Hung-Jen Liu[2,3,8,9], Der-Yuan Chen[5,6,7,10,*]

[1]Department of Medical Research, Taichung Veterans General Hospital, Taichung 407, Taiwan

[2]Rong Hsing Research Center for Translational Medicine, National Chung Hsing University, Taichung 402, Taiwan

[3]Ph.D. Program in Translational Medicine, National Chung Hsing University, Taichung 402, Taiwan

[4]Division of Allergy, Immunology and Rheumatology, Taichung Veterans General Hospital, Taichung 407, Taiwan

[5]Rheumatology and Immunology Center, China Medical University Hospital, Taichung 404, Taiwan

[6]Translational Medicine Laboratory, Rheumatology and Immunology Center, China Medical University Hospital, Taichung 404, Taiwan

[7]College of Medicine, China Medical University, Taichung 404, Taiwan

[8]Institute of Molecular Biology, National Chung Hsing University, Taichung 402, Taiwan

[9]The iEGG and Animal Biotechnology Center, National Chung Hsing University, Taichung 402, Taiwan

[10]Institute of Medicine, Chung Shan Medical University Hospital, Taichung 402, Taiwan

[§]These authors contributed equally to this work.

[*]**Corresponding author and address reprint requests:**

Dr. Der-Yuan Chen, Rheumatology and Immunology Center, Department of Medicine, China Medical University Hospital, Taichung, Taiwan.

Address: No. 2, Yude Road, Taichung, 40447, Taiwan.

Tel.: 886-4-22052121, extension 4628; Fax: 886-4-22073812

Email: dychen1957@gmail.com

Supplementary Table S1 Supplementary Materials

Reagent or Resource	Source	Identifier (Cat No.)
Antibodies		
biotinylated mouse-anti-human 8-OHdG antibody	StressMarq Biosciences	SMC-15
Mouse anti-β-actin antibodies	Santa Cruz	sc-47778
Mouse anti-CD9 antibodies	Abcam	ab58989
Mouse anti-CD66b antibodies	Bio-Rad Laboratories	MCA216T
Mouse anti-CD81 antibodies	Abcam	ab79559
Mouse anti-myeloperoxidase antibodies	Santa Cruz	sc-52707
Rabbit anti-citrullinated Histone 3 antibody	Abcam	ab5103
Rabbit anti-pAkt antibody	Cell Signaling Technology	#4060
Rabbit anti-Akt antibody	Cell Signaling Technology	#4691
Rabbit anti-CD63 antibodies	Abcam	ab68418
Rabbit anti-pERK antibody	Cell Signaling Technology	#4370
Rabbit anti-ERK antibody	Cell Signaling Technology	#4695
Rabbit anti-IL-18 antibody	R&D	MAB91243
Rabbit anti-NLRP3 antibody	Cell Signaling Technology	#15101
Alexa Fluor® 647 Goat anti-mouse IgG (H+L)	Thermo Fisher Scientific	A21235
Alexa Fluor® 647 Goat anti-rabbit IgG (H+L)	Thermo Fisher Scientific	A21244
Anti-mouse IgG, HRP-linked Antibody	Cell Signaling Technology	#7076
Anti-rabbit IgG, HRP-linked antibody	Cell Signaling Technology	#7074
Chemicals, Peptides, and Recombinant Proteins		
A23187 calcium ionophore	Sigma-Aldrich	C7522
BAPTA-AM	Sigma-Aldrich	A1076
CpG	InvivoGen	tlrl-2395

Continued		
dimethyl sulfoxide	Sigma-Aldrich	D8418
diphenyleneiodonium	Sigma-Aldrich	D2926
Fluo 4-AM	Thermo Fisher Scientific	F14217
JC-1	Abcam	ab113850
Mito-ID	Enzo Life Sciences	ENZ-51018
MitoSOX^{TM} Red	Thermo Fisher Scientific	M36008
MitoTEMPO	Sigma-Aldrich	SML0737
phorbol myristate acetate	Sigma-Aldrich	P1585
Quant-iT PicoGreen dsDNA Assay kit	Thermo Fisher Scientific	P7589
Recombinant Human IL-1β	PEPROTECH	200-01B
Recombinant Human IL-18	InvivoGen	rcyec-hil18
Sytox Green	Thermo Fisher Scientific	S7020
Tumor necrosis factor (TNF)-α	PEPROTECH	300-01A
Trizol reagent	Thermo Fisher Scientific	15596018
Others		
Lipofectamine RNAiMAX Transfection Reagent	Thermo Fisher Scientific	13778150
has-miR-223-3p miRNA mimic	Thermo Fisher Scientific	MC12301
miRNA mimic Negative Control	Thermo Fisher Scientific	4464058
has-miR-223-3p miRNA inhibitor	Thermo Fisher Scientific	MH12301
miRNA inhibitor Negative Control	Thermo Fisher Scientific	4464084
has-miR-223-3p TaqMan MicroRNA Assays	Thermo Fisher Scientific	002295

Supplementary Figure S1

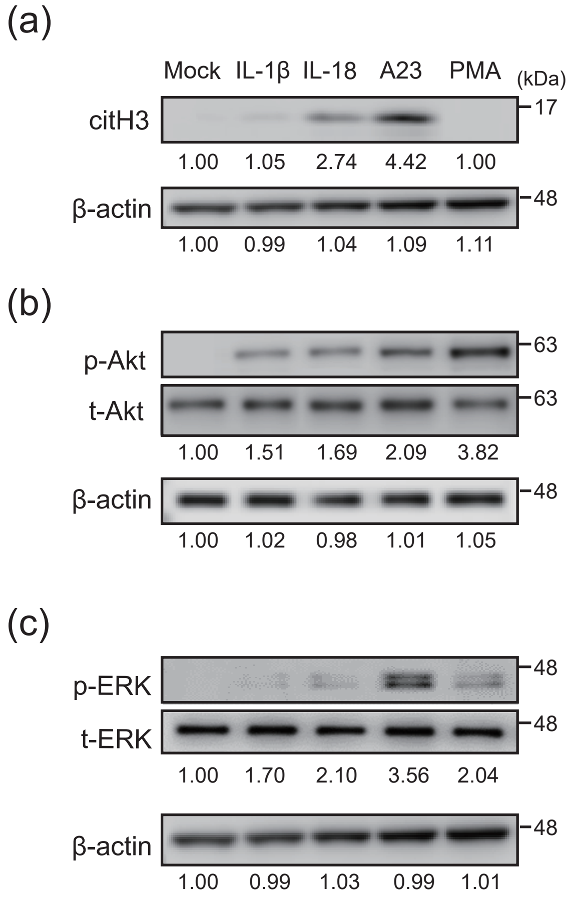

Figure S1 Increased levels of (a) citrullinated histone H3 (citH3), (b)Akt, and (c) ERK activation in dHL-60 cells with IL-18 treatment.

Supplementary Figure S2

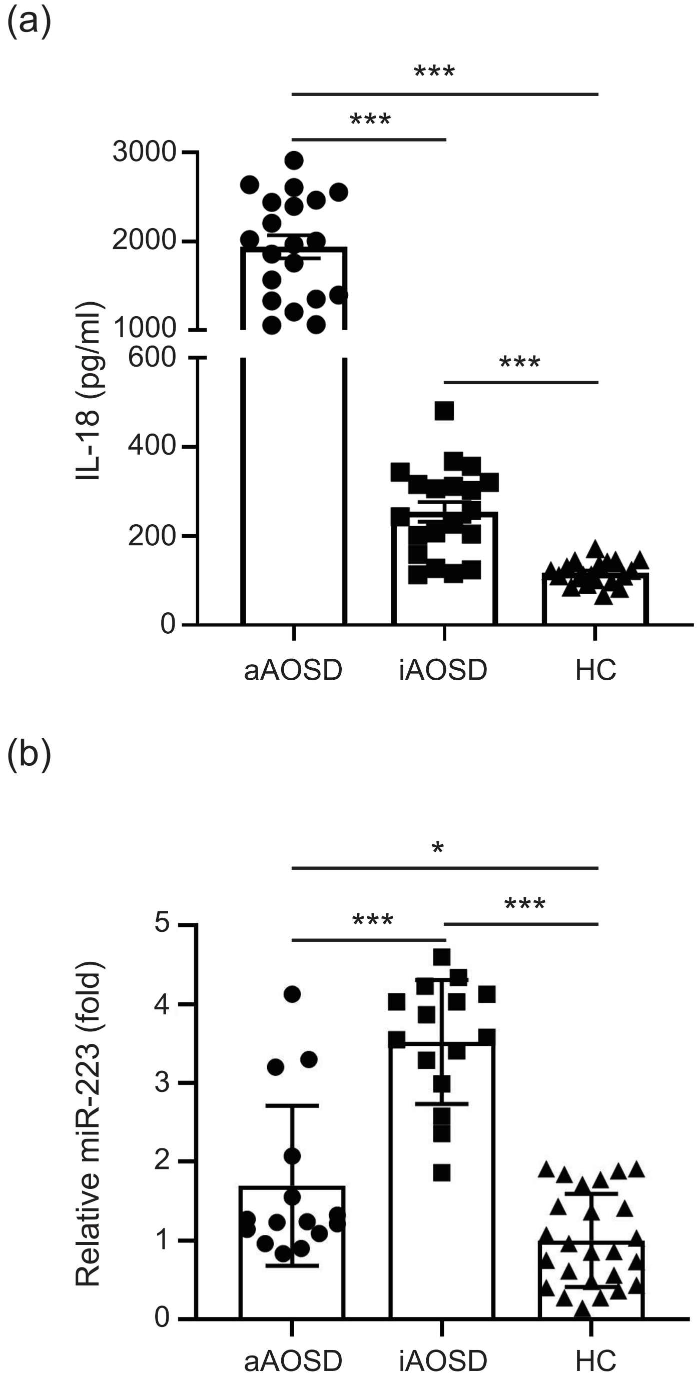

Supplementary Figure S2 The (a) circulating levels of IL-18 in sera and (b) miR-223 in peripheral blood mononuclear cells (PBMCs) from patients with active AOSD (aAOSD), inactive AOSD (iAOSD), and healthy controls (HC). Data are presented as the mean ± SD. **P*<0.05, ****P*<0.005.

Supplementary Figure S3

(a)

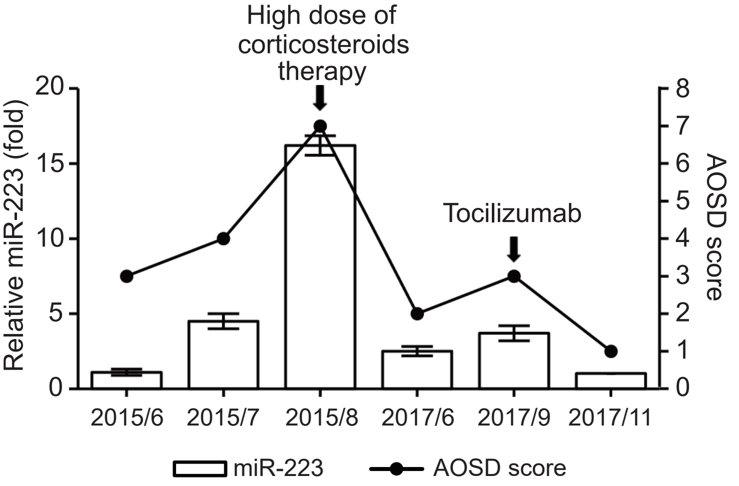

(b)

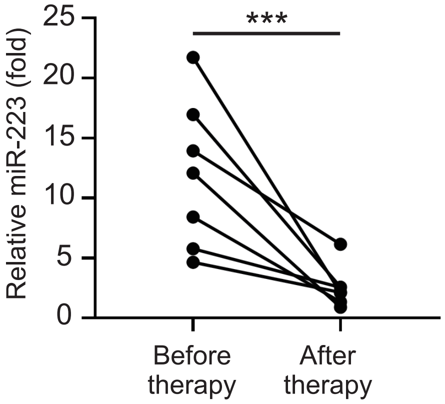

Supplementary Figure S3 (a) The dynamic of AOSD activity scores and circulating miR-223 expression levels in an AOSD patient during therapy period. (b) Decreases in miR-223 expression levels paralleled the clinical remission in AOSD patients after therapy. All experiments were performed in triplicate, and data are presented as the mean ± SD. ****P*<0.005.

Supplementary Figure S4

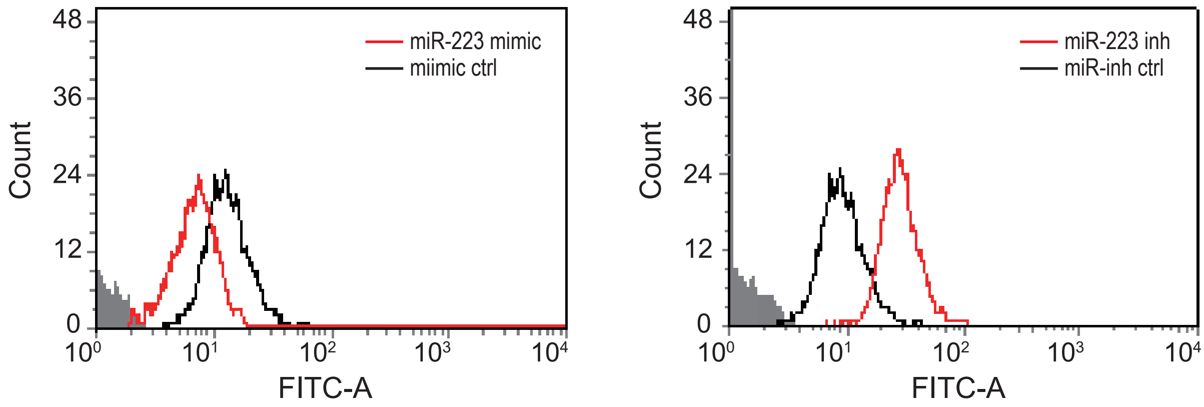

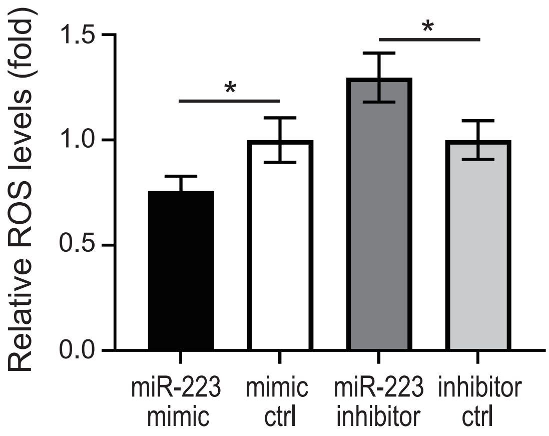

Supplementary Figure S4 MiR-223 inhibited IL-18-induced an elevated cytosolic ROS production in dHL-60 cells by using DCFDA fluorescent dye staining and quantified by flow cytometry.

Supplementary Figure S5

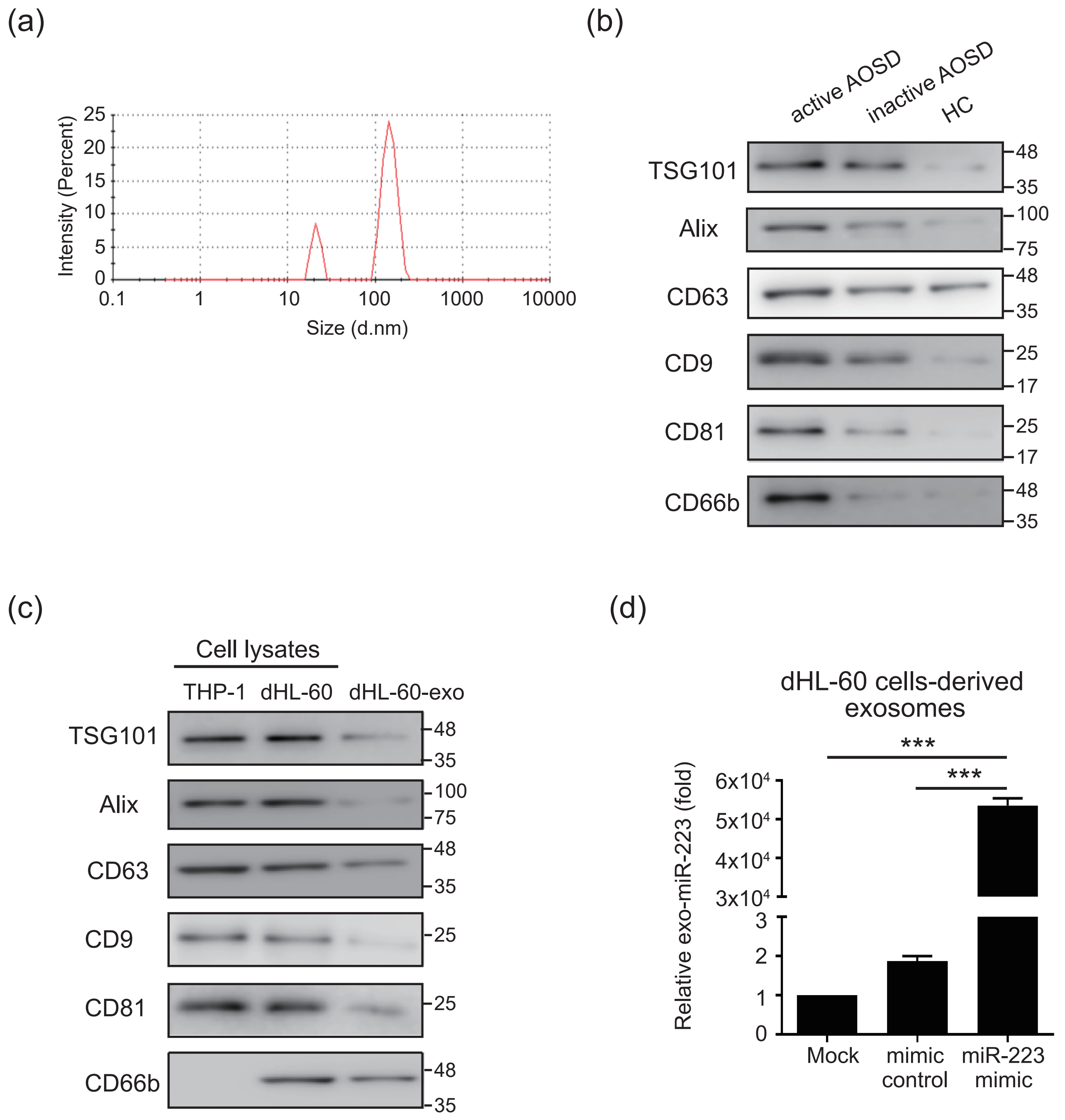

Supplementary Figure S5 (a) The proportion and particle size distribution of extracellular vesicles were extracted from sera of AOSD patient. (b and c) Expression of exosomal protein TSG101, Alix, CD63, CD9, CD81, and neutrophil surface marker CD66b in (b) patient with active AOSD, inactive AOSD, healthy control, and (c) dHL-60 cells–derived exosome was analyzed using immunoblotting. (d) The relative levels of encapsulated miR-223 were determined using QRT-PCR. All experiments were performed in triplicate, and data are presented as mean±SD. ****P*<0.0001 was determine using student's t-test

Supplementary Figure S6

(a)

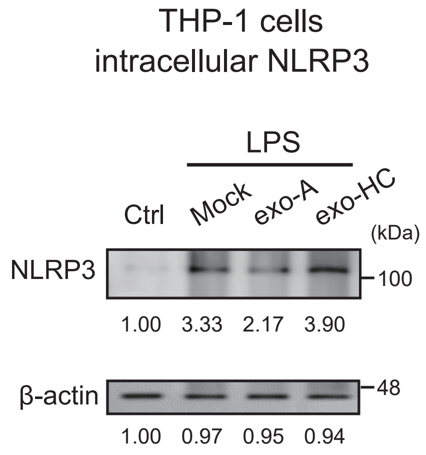

(b)

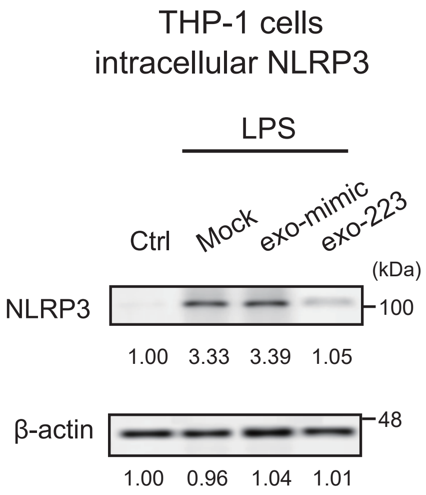

Supplementary Figure S6 THP-1–derived macrophages were pretreated with LPS (1μg/ml) for 1 h to activate NLRP3, then co-cultured with (a) the purified exosomes from active AOSD patients or HC; (b) dHL-60 cell-derived exosomes were loaded with mimic miR-223, or control for 24 h, respectively. The levels of intracellular NLRP3 were measured by using immunoblotting.

Supplementary Figure S7

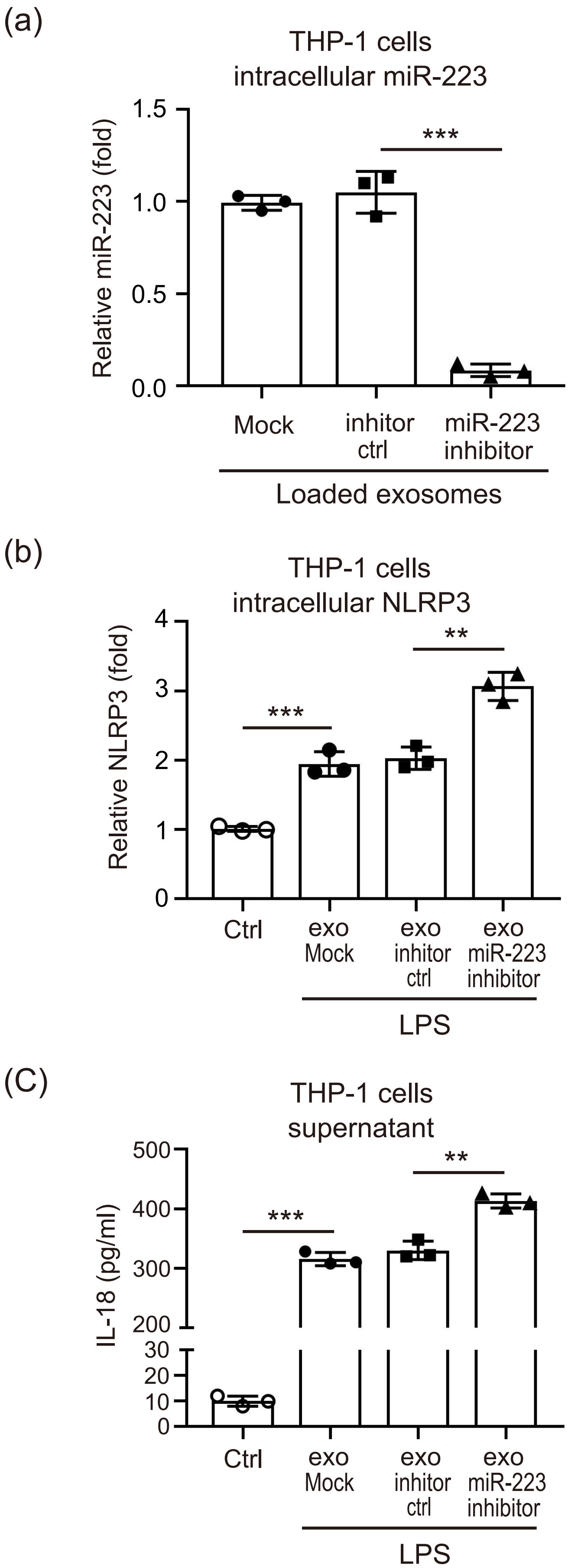

Supplementary Figure S7 MiR-223 in neutrophil-derived exosomes contribute to the inhibition of IL-18 secretion in macrophages. dHL-60 cell-derived exosomes were loaded with miR-223 inhibitor, or control. THP-1–derived macrophages were pretreated with LPS (1μg/ml) for 1 h to activate NLRP3, then co-cultured with the indicated loaded exosomes for 24 h, respectively. The levels of (a) intracellular miR-223, (b) intracellular NLRP3, and (c) secretory IL-18 were measured by using QRT-PCR, and ELISA, respectively. All experiments were performed in triplicate, and data are presented as the mean±SD. ***P*<0.01, ****P*<0.0001 was determine using student's t-test.

Supplementary information

Images of full-length gels and blots

Supplementary Fig. S1

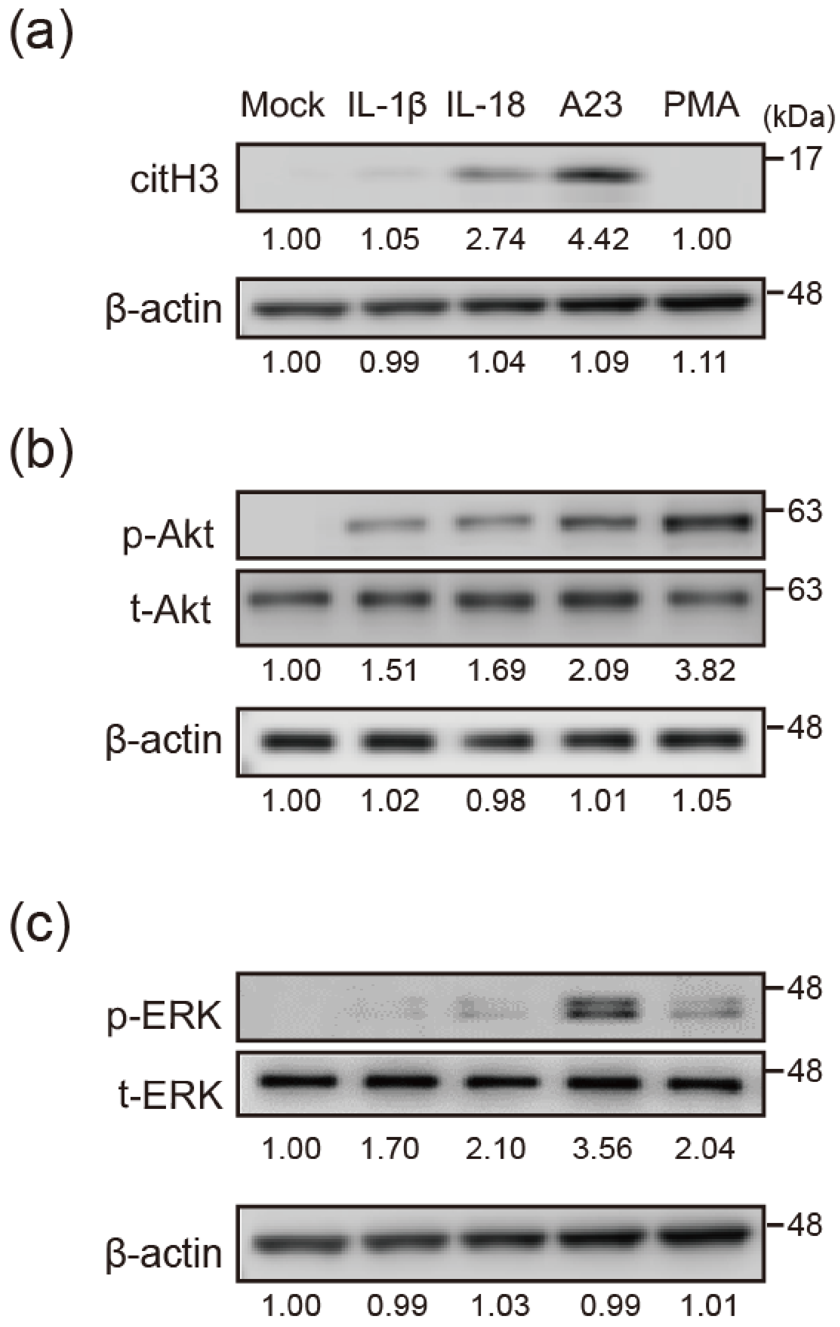

citH3 (15 kDa)

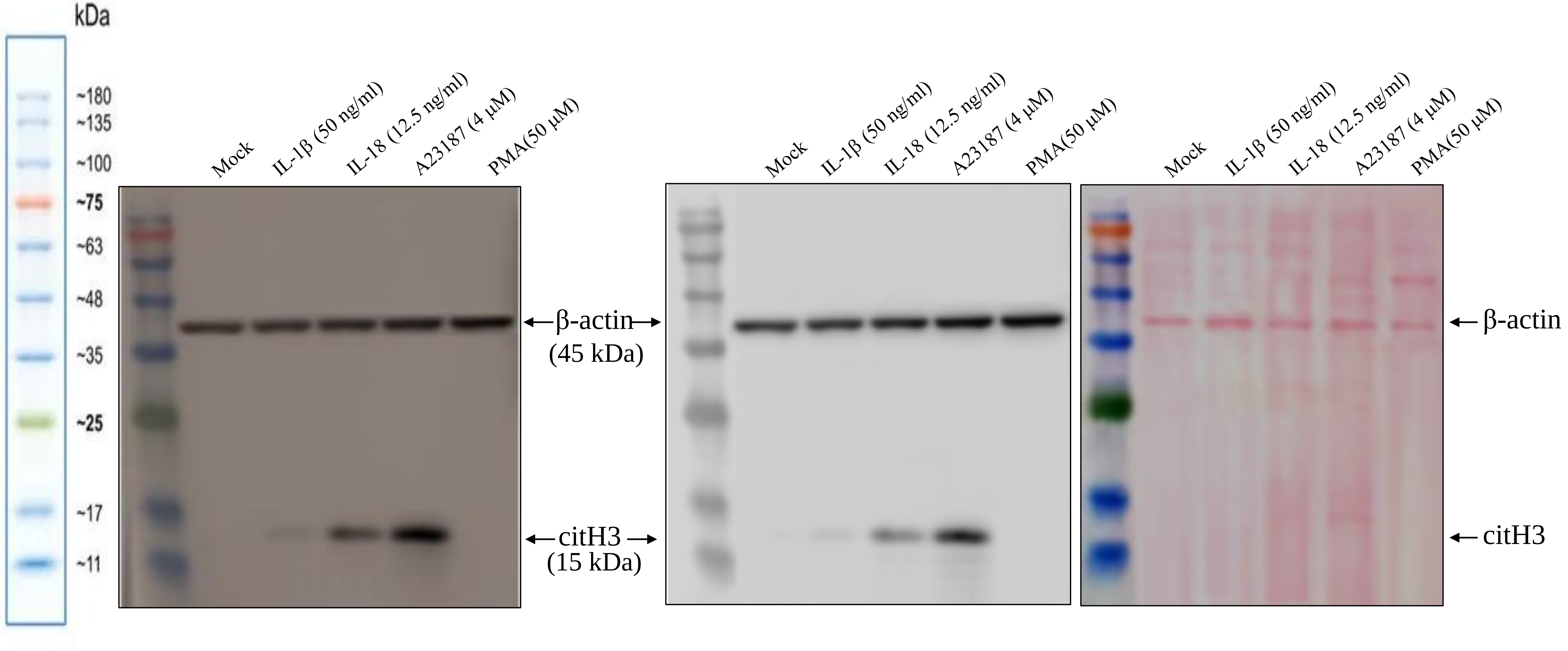

kDa
~180
~135
~100
~75
~63
~48
~35
~25
~17
~11
Mock
IL-1β (50 ng/ml)
IL-18 (12.5 ng/ml)
A23187 (4 μM)
PMA(50 μM)
β-actin
(45 kDa)
citH3
(15 kDa)
Mock
IL-1β (50 ng/ml)
IL-18 (12.5 ng/ml)
A23187 (4 μM)
PMA(50 μM)
Mock
IL-1β (50 ng/ml)
IL-18 (12.5 ng/ml)
A23187 (4 μM)
PMA(50 μM)
β-actin
citH3

p-Akt (60 kDa)

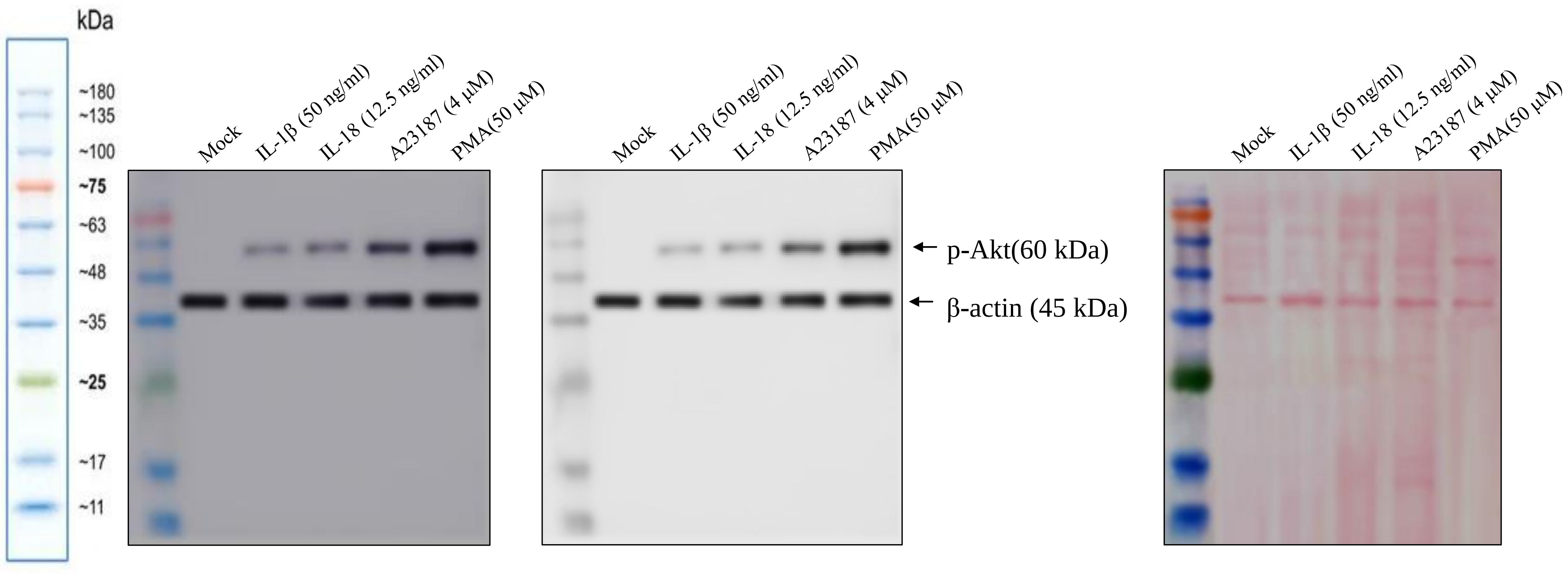

kDa
~180
~135
~100
~75
~63
~48
~35
~25
~17
~11
Mock
IL-1β (50 ng/ml)
IL-18 (12.5 ng/ml)
A23187 (4 μM)
PMA(50 μM)
p-Akt(60 kDa)
β-actin (45 kDa)

t-Akt (60 kDa)

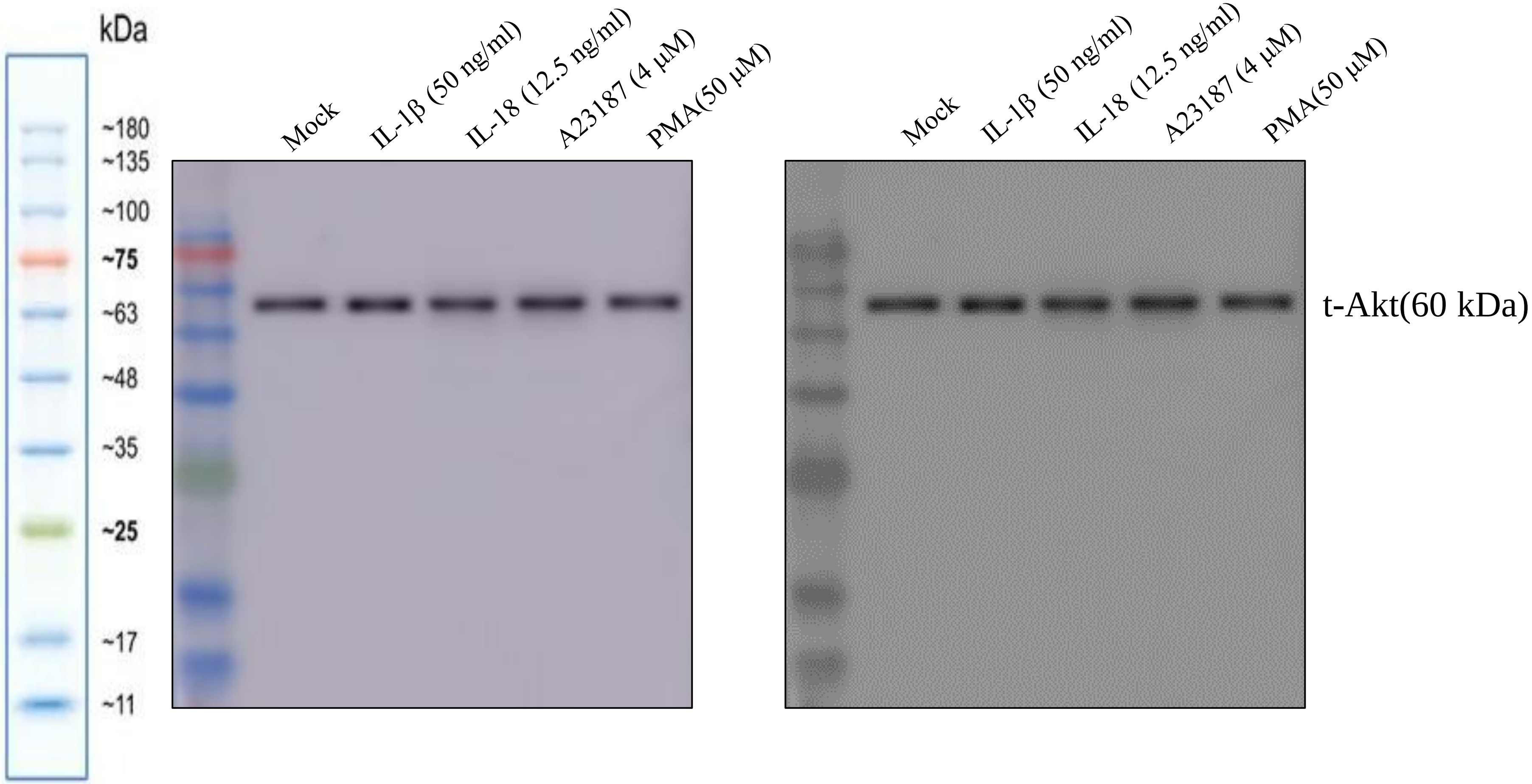

kDa
~180
~135
~100
~75
~63
~48
~35
~25
~17
~11
Mock
IL-1β (50 ng/ml)
IL-18 (12.5 ng/ml)
A23187 (4 μM)
PMA(50 μM)
Mock
IL-1β (50 ng/ml)
IL-18 (12.5 ng/ml)
A23187 (4 μM)
PMA(50 μM)
t-Akt(60 kDa)

p-ERK (44 kDa)

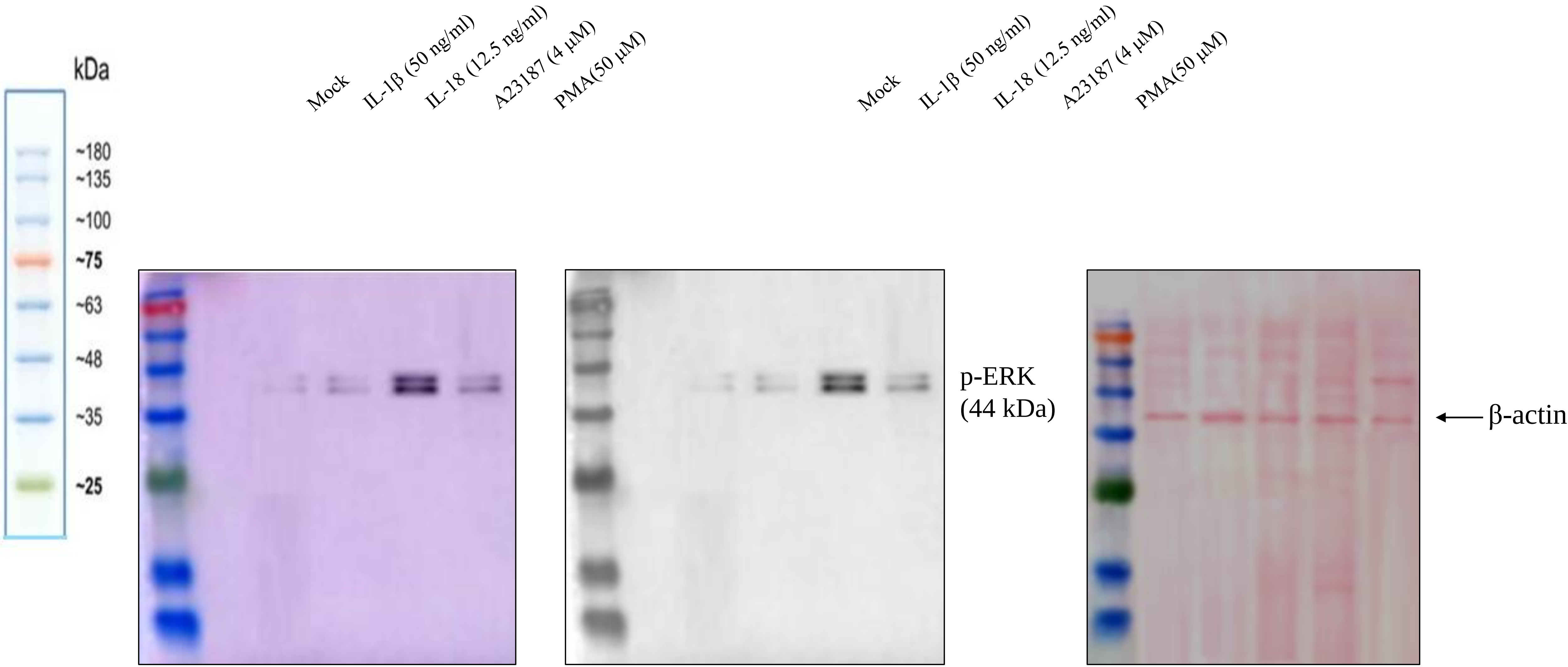

kDa
~180
~135
~100
~75
~63
~48
~35
~25
Mock
IL-1β (50 ng/ml)
IL-18 (12.5 ng/ml)
A23187 (4 μM)
PMA(50 μM)
Mock
IL-1β (50 ng/ml)
IL-18 (12.5 ng/ml)
A23187 (4 μM)
PMA(50 μM)
p-ERK
(44 kDa)
β-actin

t-ERK (44 kDa)

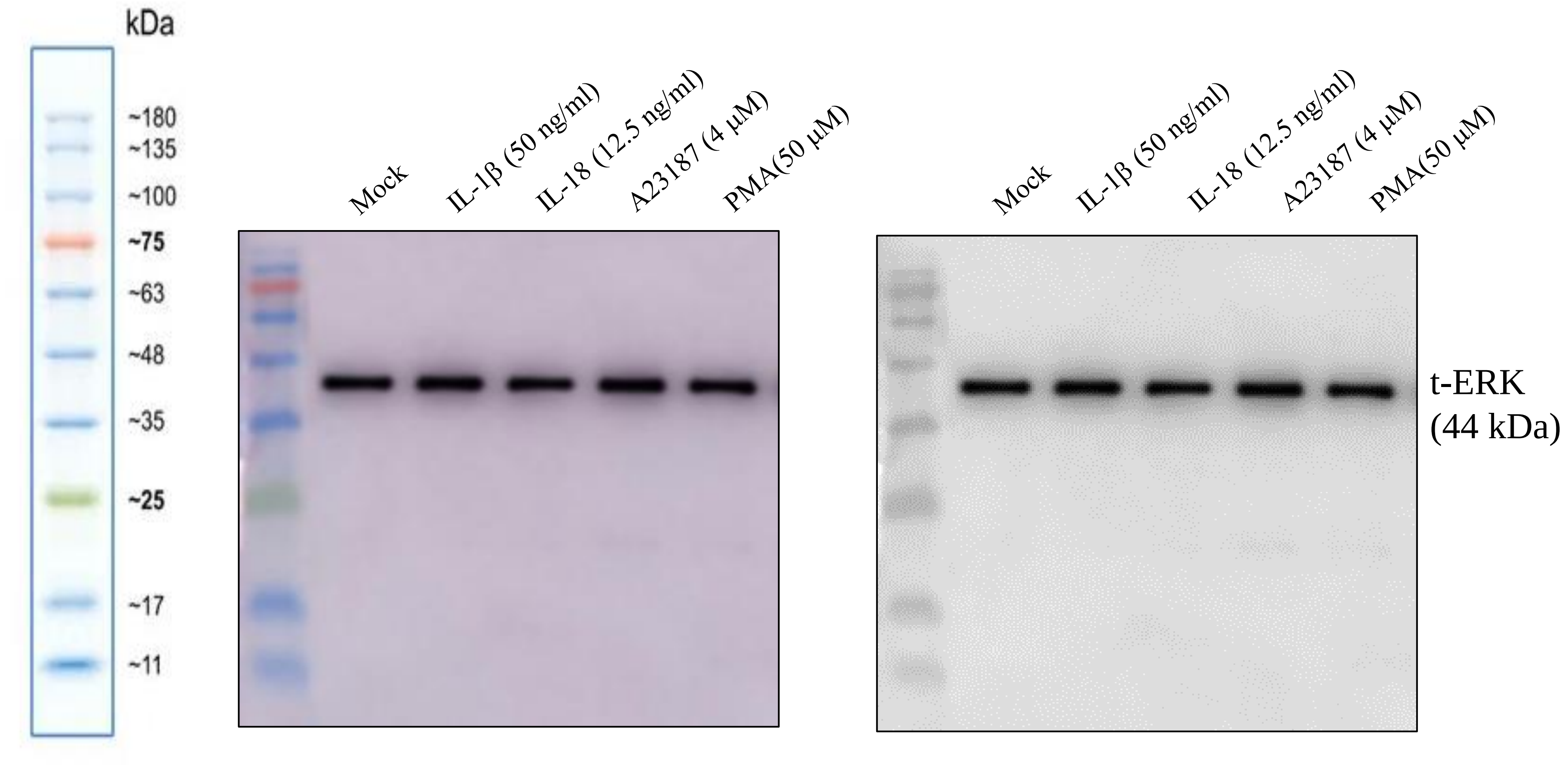
kDa
~180
~135
~100
~75
~63
~48
~35
~25
~17
~11
Mock
IL-1β (50 ng/ml)
IL-18 (12.5 ng/ml)
A23187 (4 μM)
PMA(50 μM)
Mock
IL-1β (50 ng/ml)
IL-18 (12.5 ng/ml)
A23187 (4 μM)
PMA(50 μM)
t-ERK
(44 kDa)

β-actin (45 kDa)

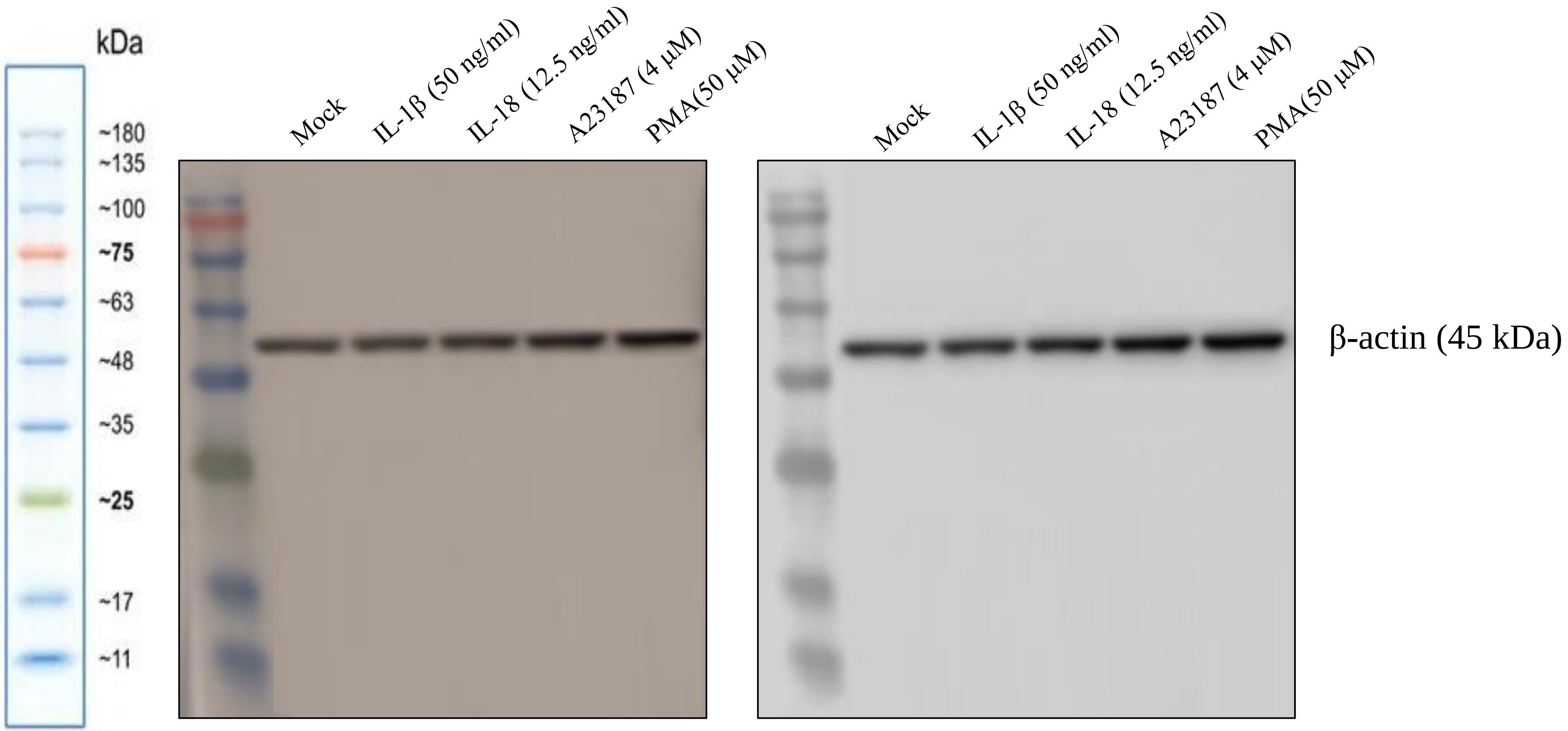

Supplementary Fig. S5b

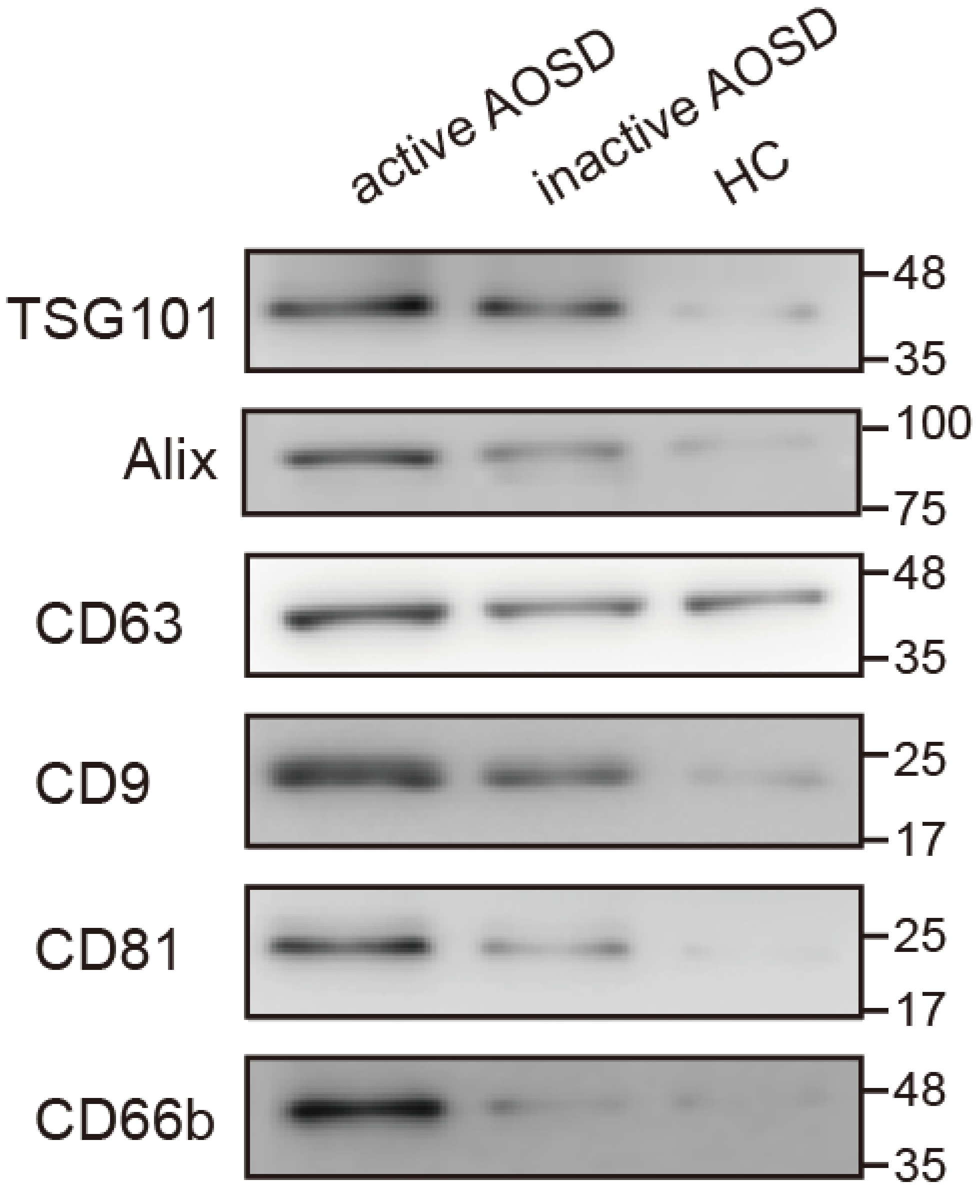

Tsg101 (45 kDa)

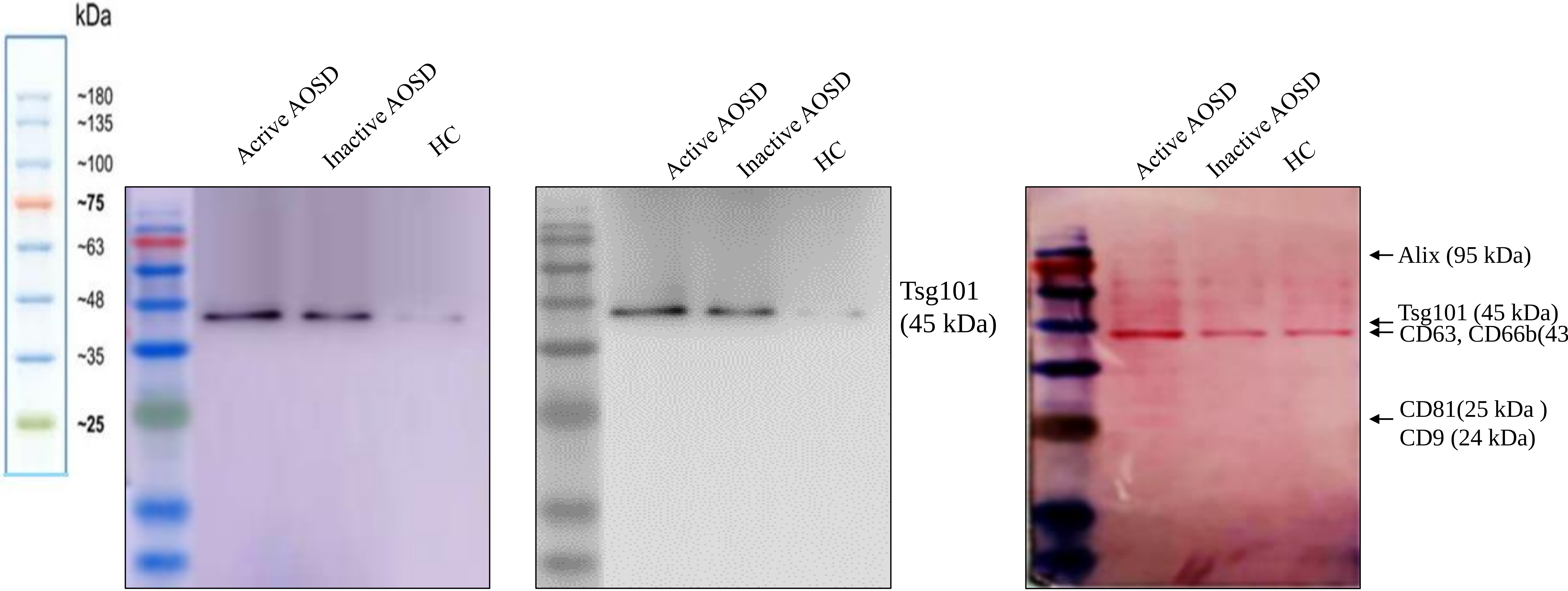

Alix (95 kDa)

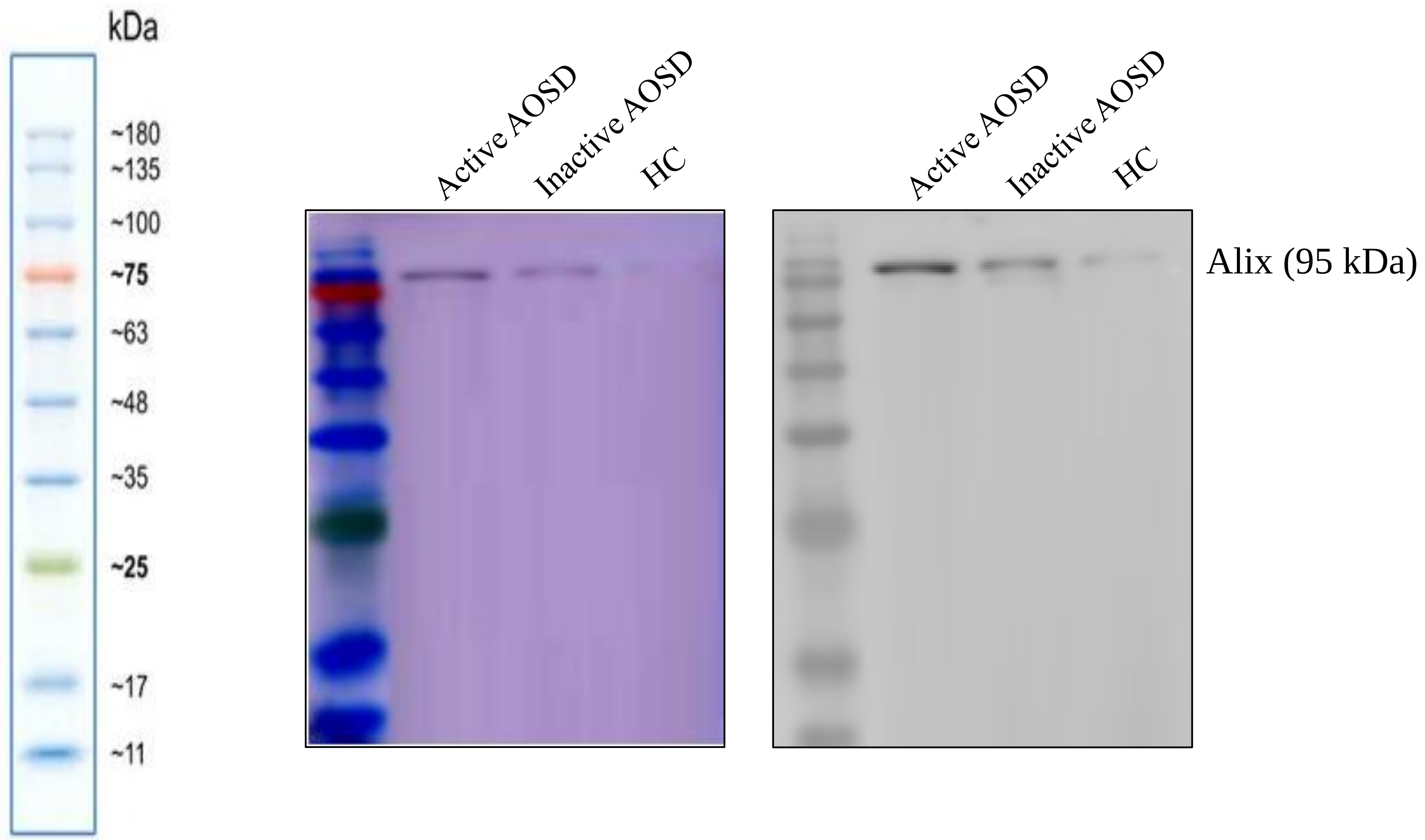

kDa
~180
~135
~100
~75
~63
~48
~35
~25
~17
~11
Active AOSD
Inactive AOSD
HC
Active AOSD
Inactive AOSD
HC
Alix (95 kDa)

CD63 (43 kDa)

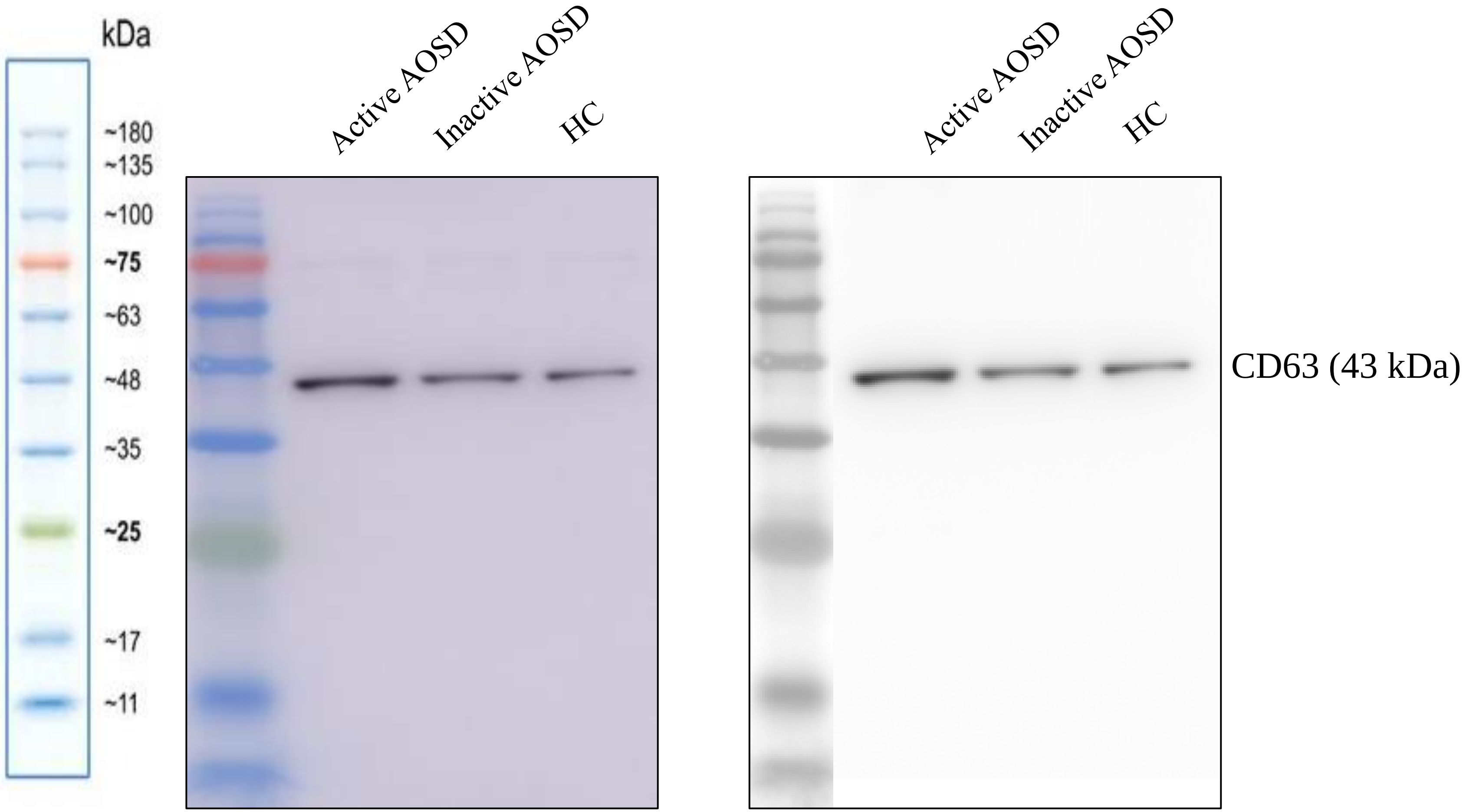
kDa
~180
~135
~100
~75
~63
~48
~35
~25
~17
~11
Active AOSD
Inactive AOSD
HC
Active AOSD
Inactive AOSD
HC
CD63 (43 kDa)

CD9 (24 kDa)

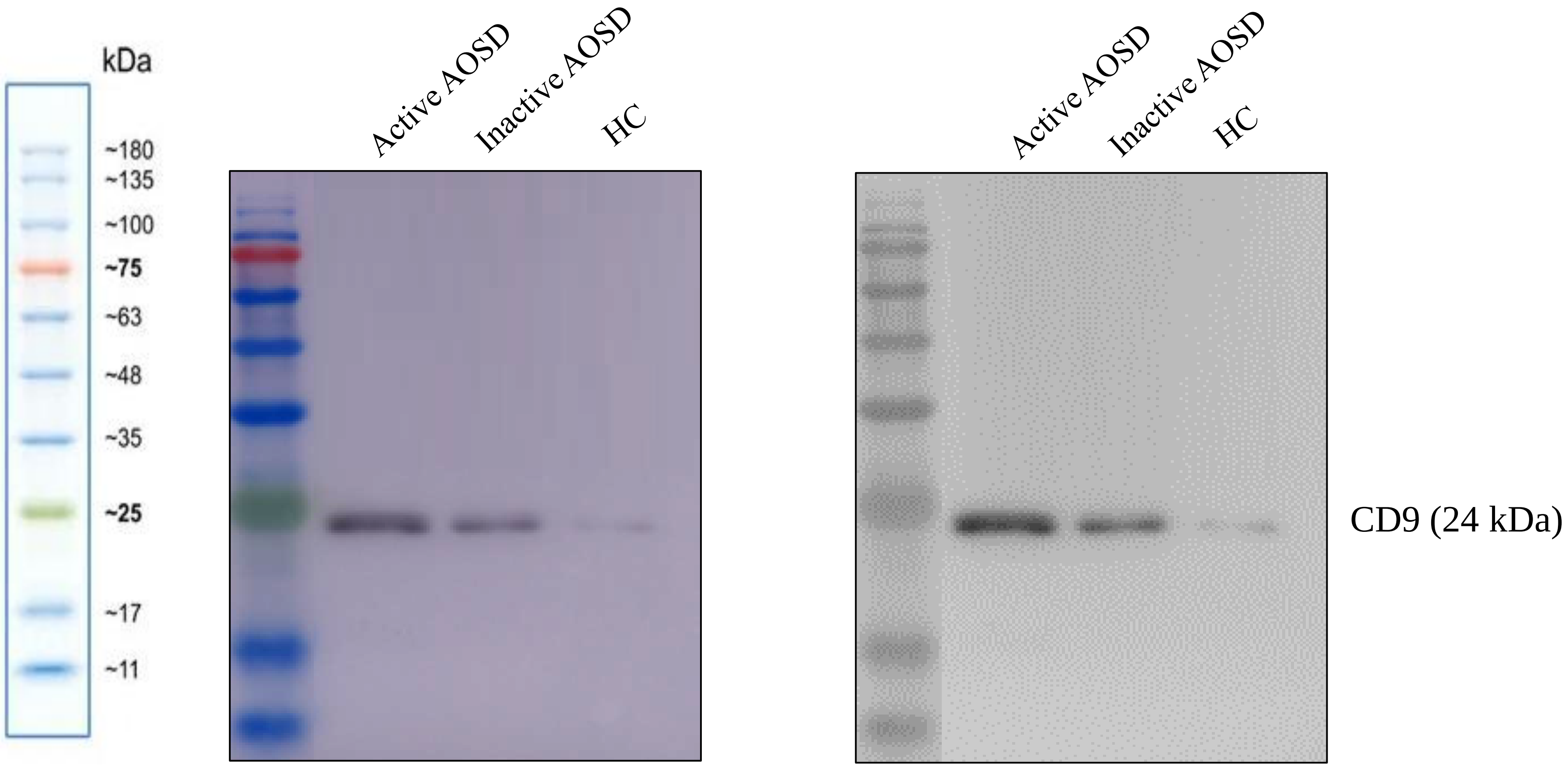
kDa
~180
~135
~100
~75
~63
~48
~35
~25
~17
~11
Active AOSD
Inactive AOSD
HC
Active AOSD
Inactive AOSD
HC
CD9 (24 kDa)

CD81 (25 kDa)

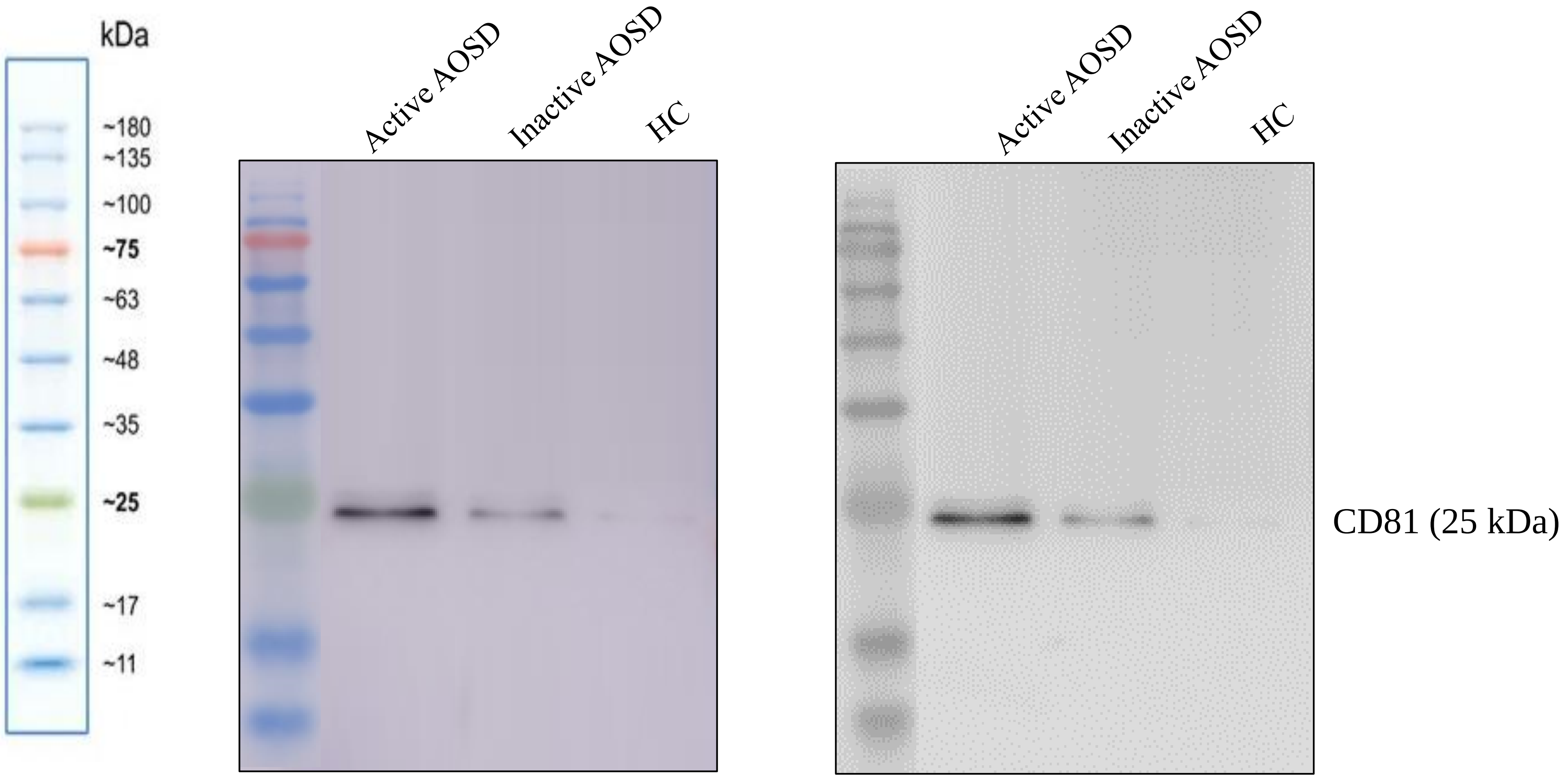
kDa
~180
~135
~100
~75
~63
~48
~35
~25
~17
~11
Active AOSD
Inactive AOSD
HC
Active AOSD
Inactive AOSD
HC
CD81 (25 kDa)

CD66b (43 kDa)

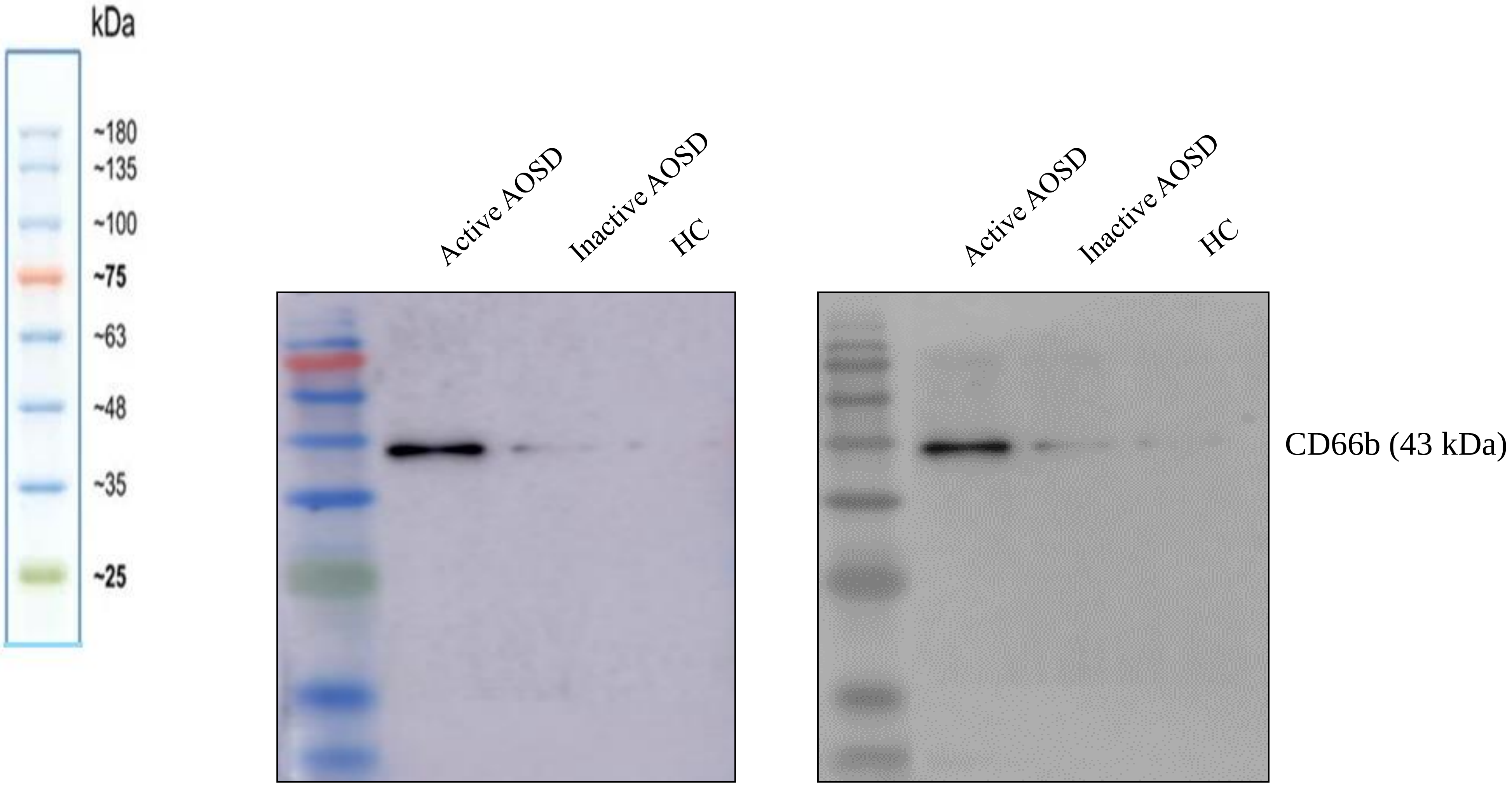

kDa
~180
~135
~100
~75
~63
~48
~35
~25
Active AOSD
Inactive AOSD
HC
Active AOSD
Inactive AOSD
HC
CD66b (43 kDa)

Supplementary Fig. S5c

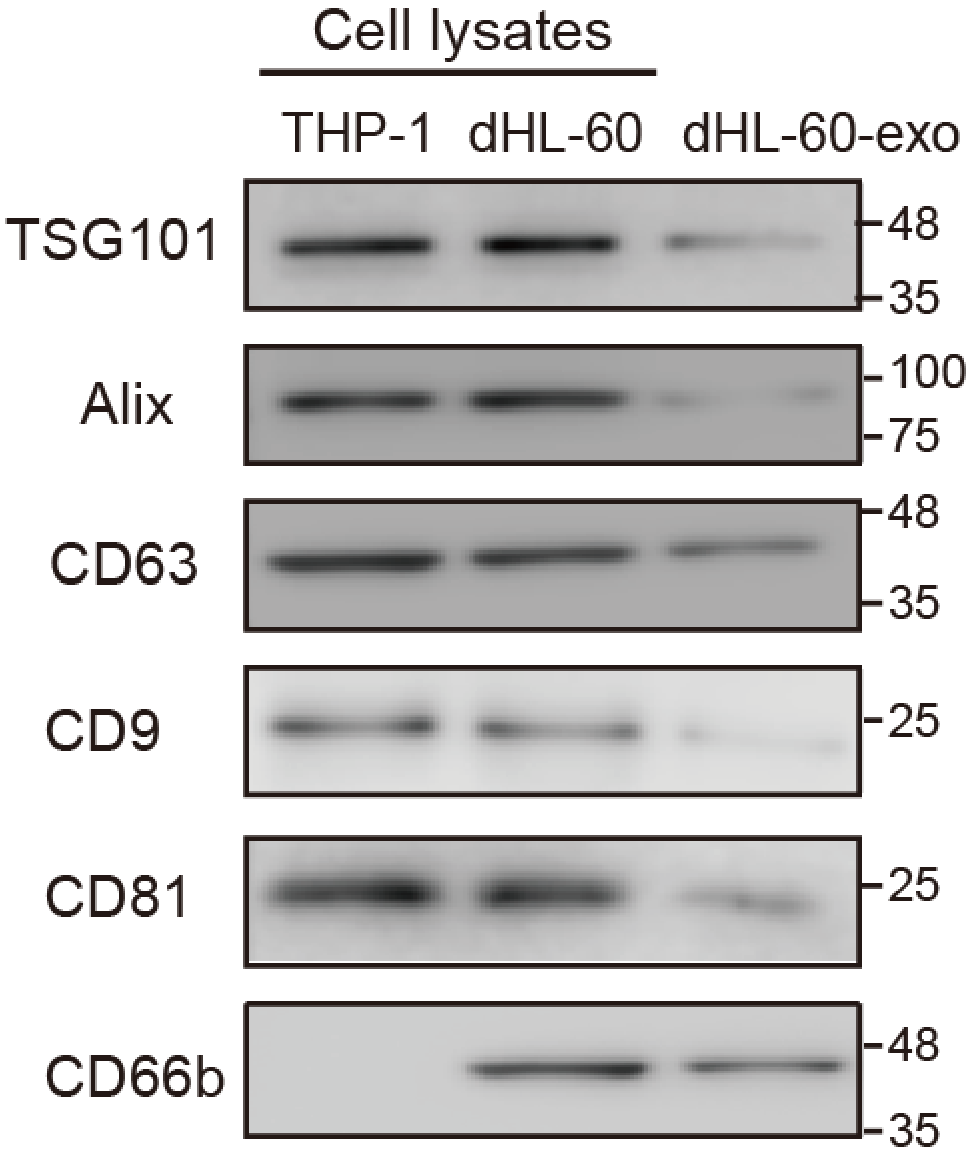

Tsg101 (45 kDa)

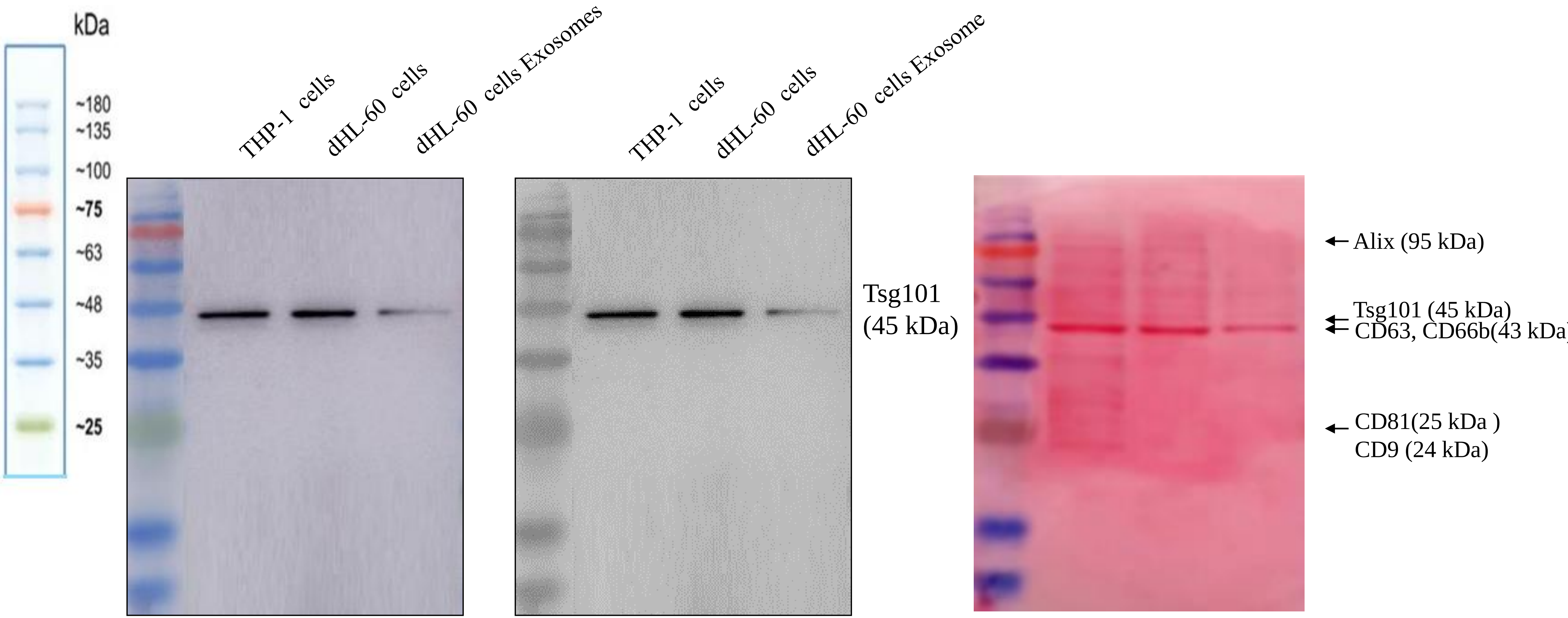

Alix (95 kDa)

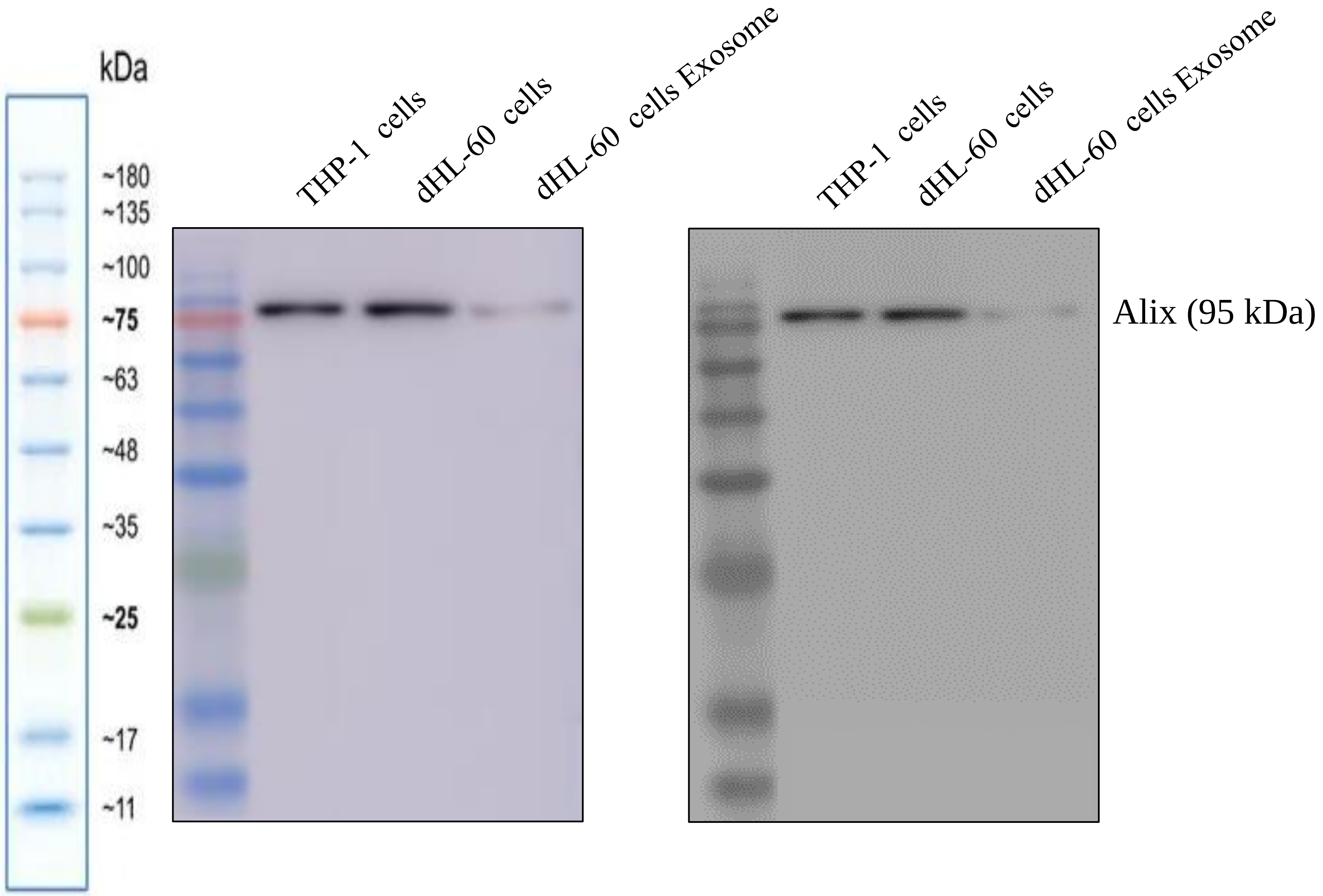
kDa
~180
~135
~100
~75
~63
~48
~35
~25
~17
~11
THP-1 cells
dHL-60 cells
dHL-60 cells Exosome
THP-1 cells
dHL-60 cells
dHL-60 cells Exosome
Alix (95 kDa)

CD63 (43 kDa)

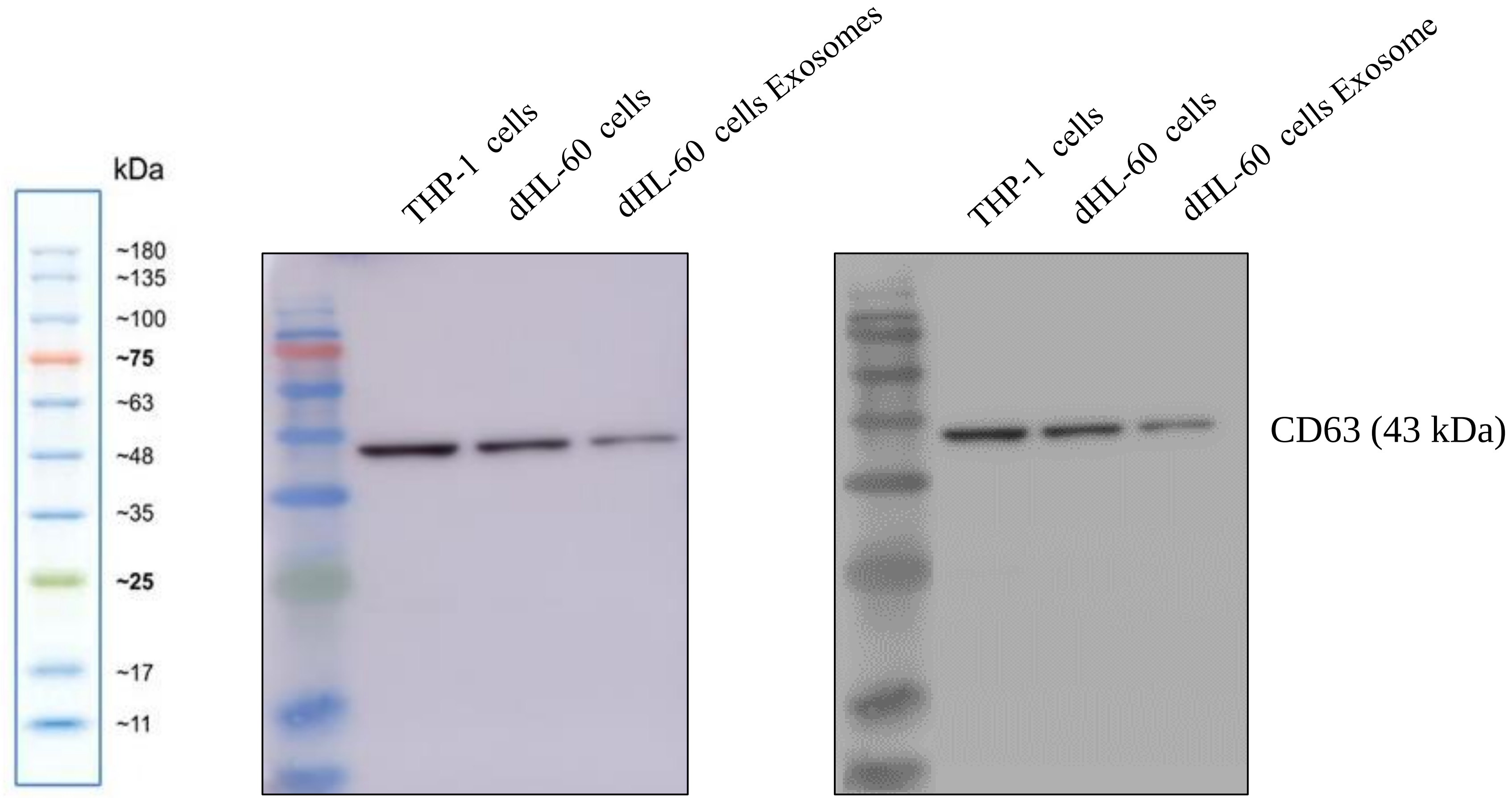
kDa
~180
~135
~100
~75
~63
~48
~35
~25
~17
~11
THP-1 cells
dHL-60 cells
dHL-60 cells Exosomes
THP-1 cells
dHL-60 cells
dHL-60 cells Exosome
CD63 (43 kDa)

CD9 (24 kDa)

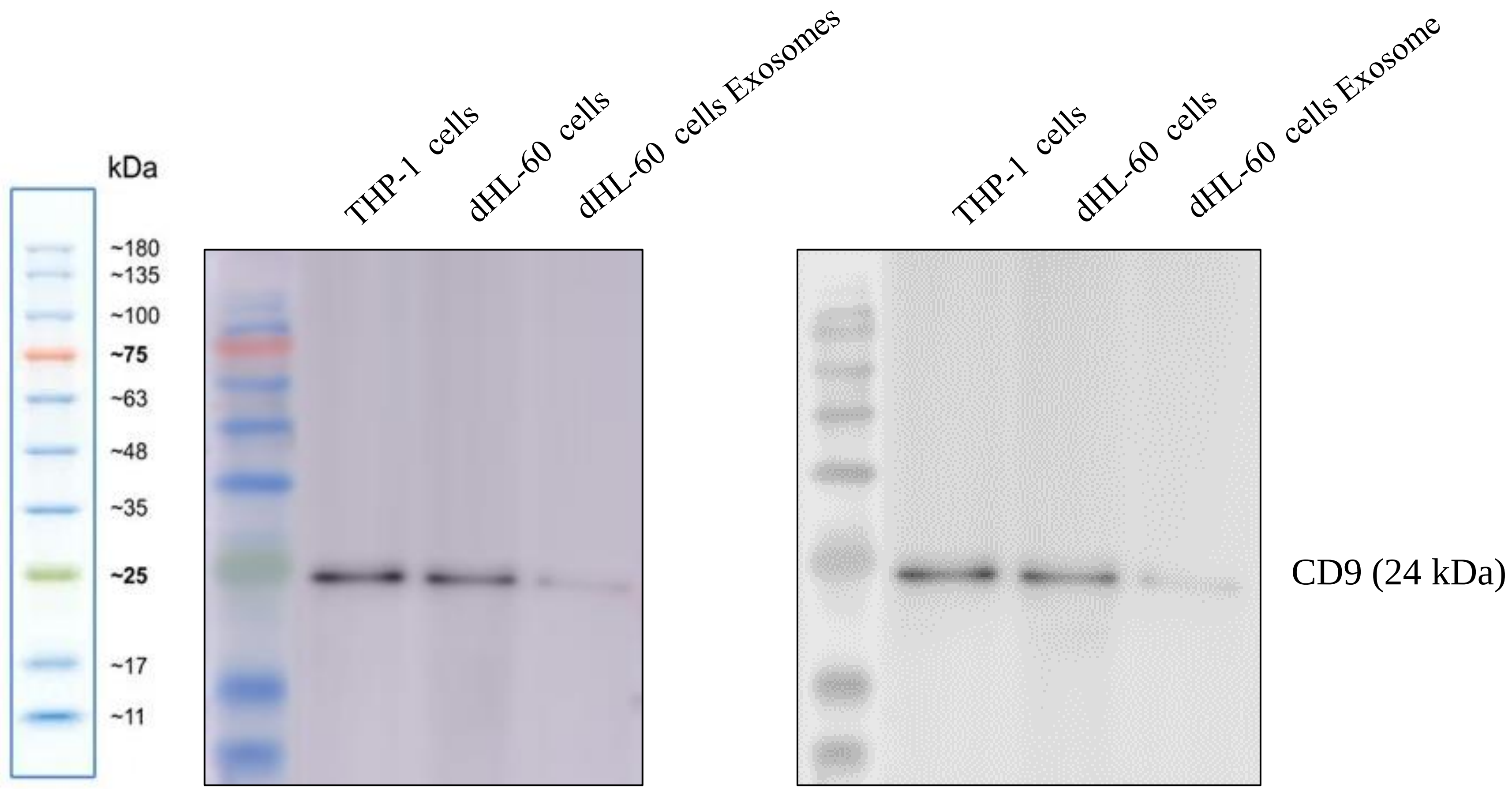
kDa
~180
~135
~100
~75
~63
~48
~35
~25
~17
~11
THP-1 cells
dHL-60 cells
dHL-60 cells Exosomes
THP-1 cells
dHL-60 cells
dHL-60 cells Exosome
CD9 (24 kDa)

CD81 (25 kDa)

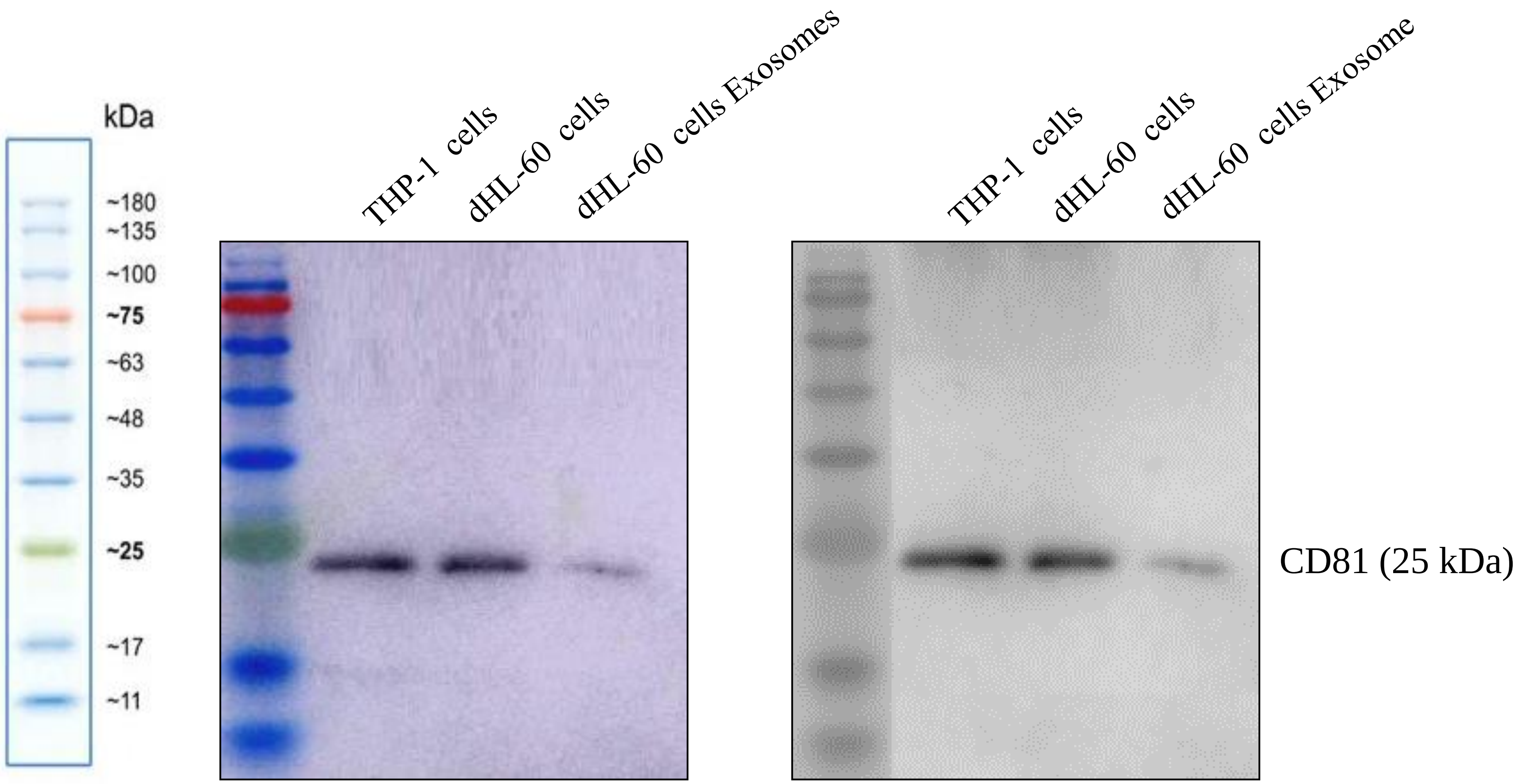

kDa
~180
~135
~100
~75
~63
~48
~35
~25
~17
~11
THP-1 cells
dHL-60 cells
dHL-60 cells Exosomes
THP-1 cells
dHL-60 cells
dHL-60 cells Exosome
CD81 (25 kDa)

CD66b (43 kDa)

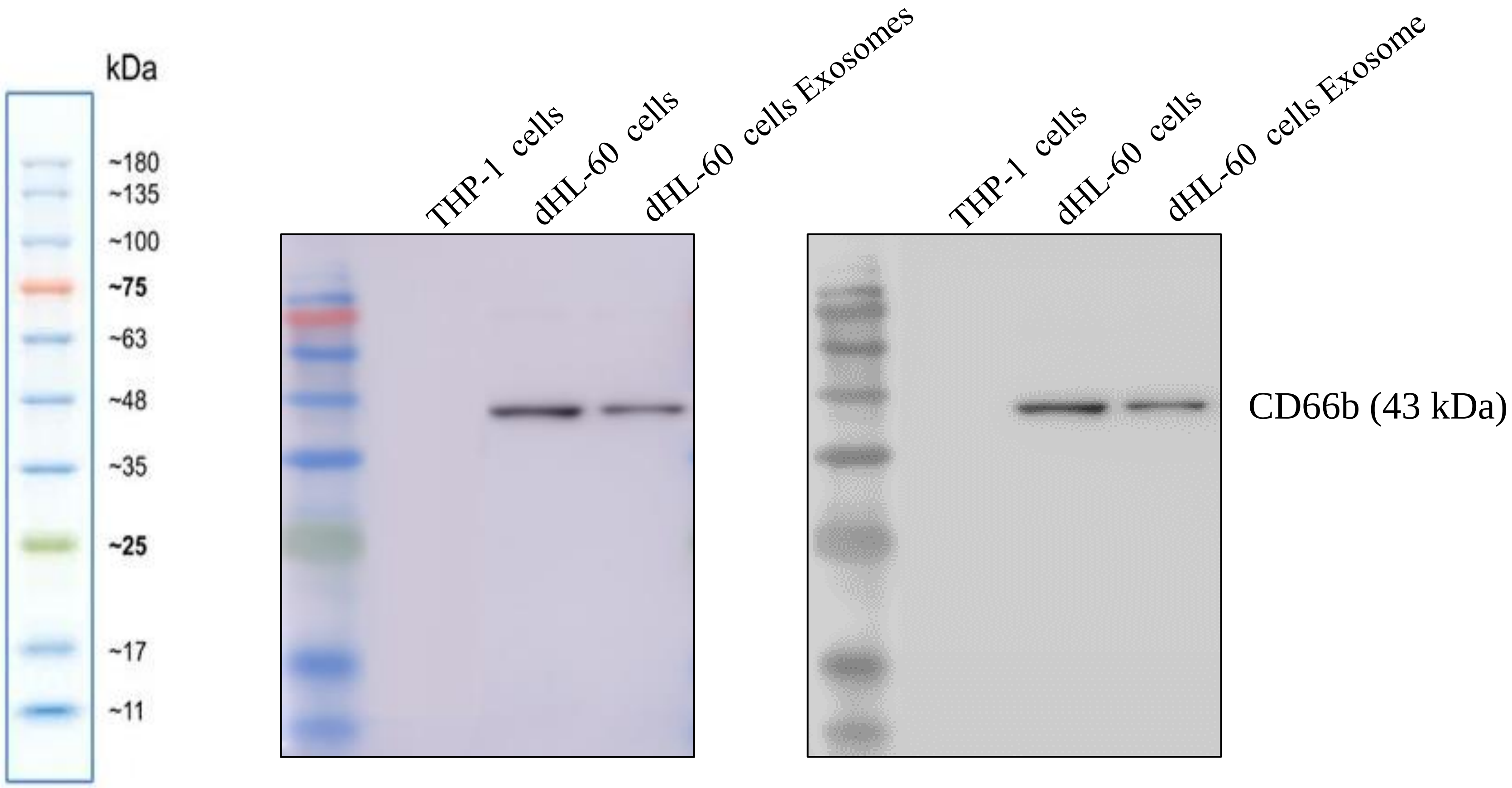

kDa
~180
~135
~100
~75
~63
~48
~35
~25
~17
~11
THP-1 cells
dHL-60 cells
dHL-60 cells Exosomes
THP-1 cells
dHL-60 cells
dHL-60 cells Exosome
CD66b (43 kDa)

Supplementary Fig. S6a

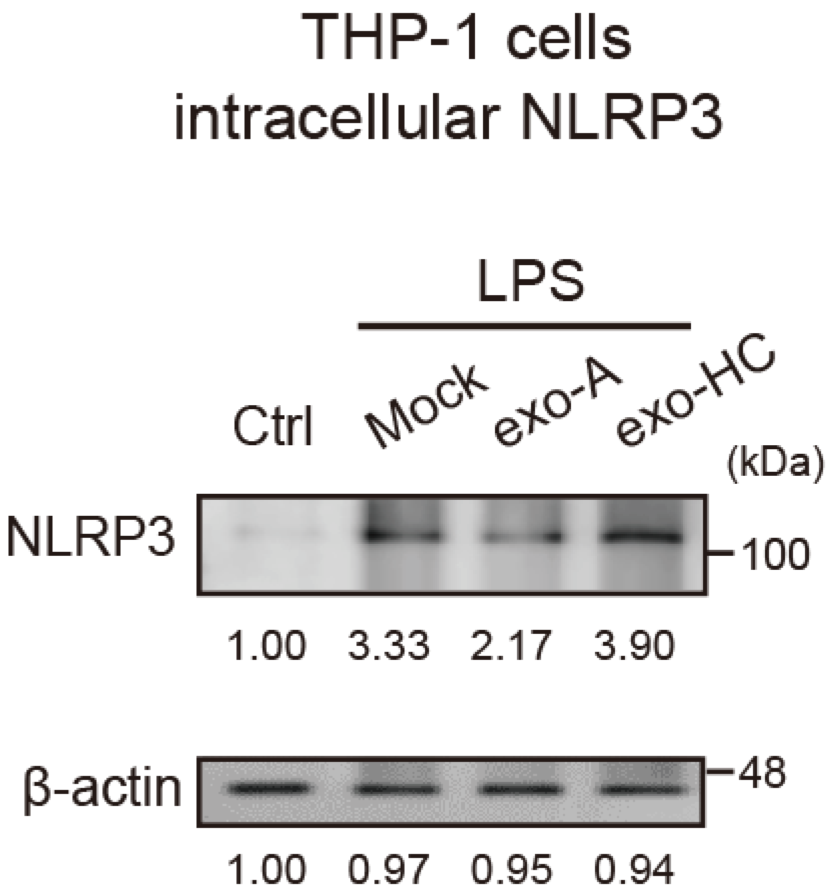

NLRP3 (110 kDa)

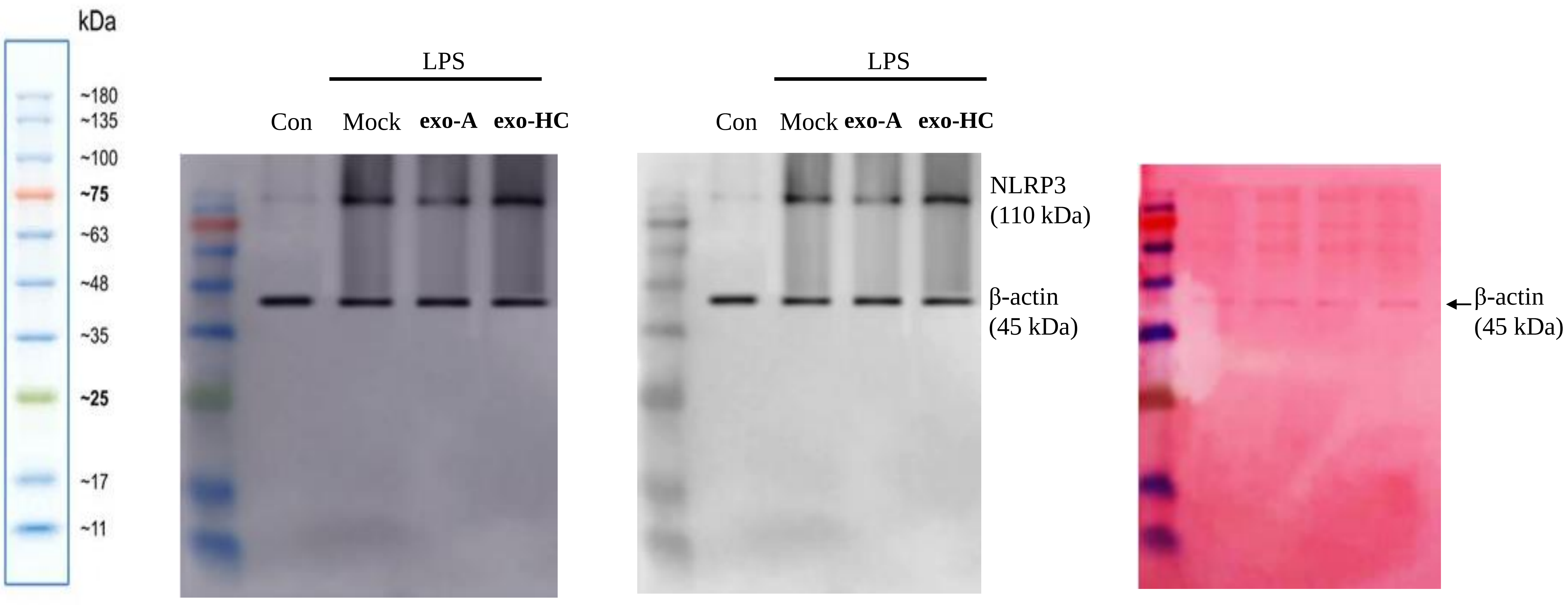

Supplementary Fig. S6b

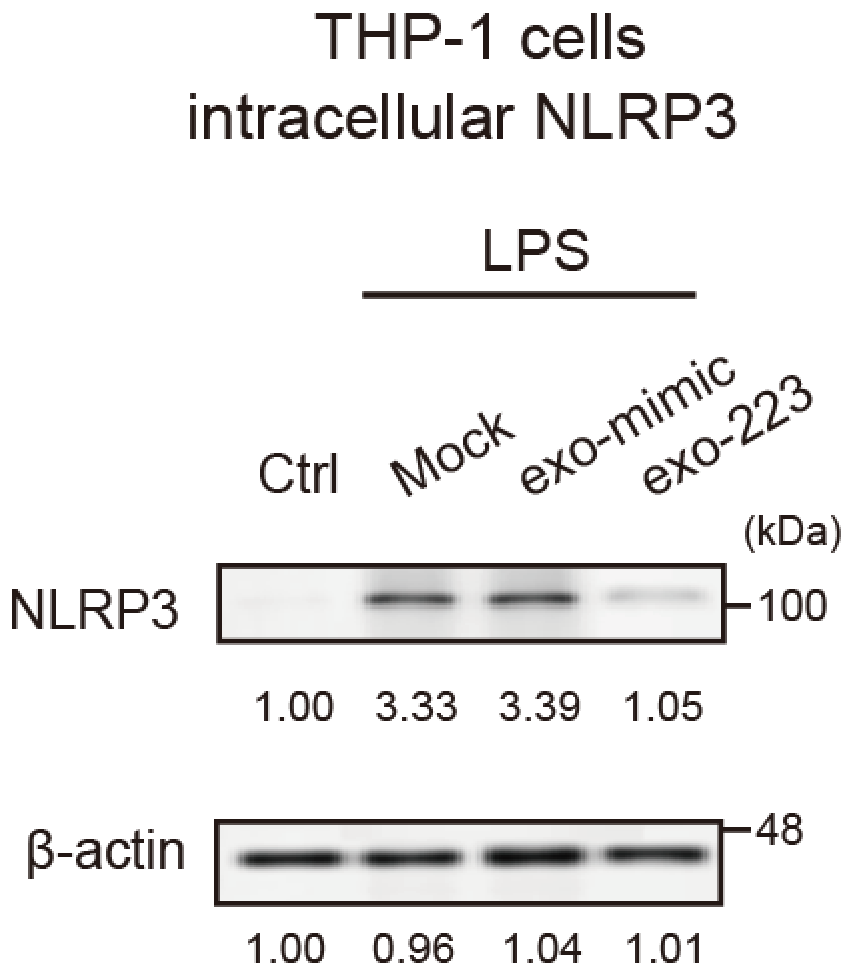

NLRP3 (110 kDa)

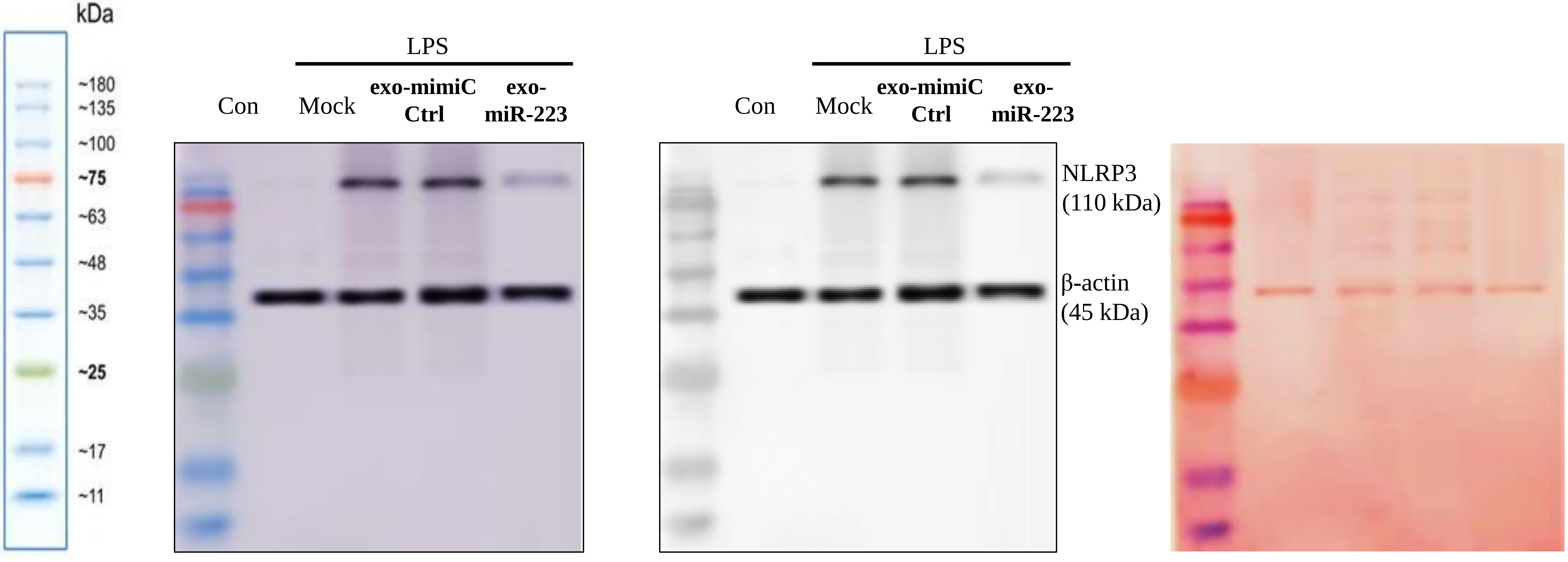

Supplement: Supplementary file 1 — Supplementary Information. [file 41598_2021_95028_MOESM1_ESM.pdf]
